# ADRA1A–Gα_q_ signalling potentiates adipocyte thermogenesis through CKB and TNAP

**DOI:** 10.1038/s42255-022-00667-w

**Published:** 2022-11-07

**Authors:** Janane F. Rahbani, Charlotte Scholtes, Damien M. Lagarde, Mohammed F. Hussain, Anna Roesler, Christien B. Dykstra, Jakub Bunk, Bozena Samborska, Shannon L. O’Brien, Emma Tripp, Alain Pacis, Anthony R. Angueira, Olivia S. Johansen, Jessica Cinkornpumin, Ishtiaque Hossain, Matthew D. Lynes, Yang Zhang, Andrew P. White, William A. Pastor, Maria Chondronikola, Labros Sidossis, Samuel Klein, Anastasia Kralli, Aaron M. Cypess, Steen B. Pedersen, Niels Jessen, Yu-Hua Tseng, Zachary Gerhart-Hines, Patrick Seale, Davide Calebiro, Vincent Giguère, Lawrence Kazak

**Affiliations:** 1 Rosalind & Morris Goodman Cancer Institute, McGill University, Montreal, Quebec Canada; 2 Department of Biochemistry, McGill University, Montreal, Quebec Canada; 3 Institute of Metabolism and Systems Research, University of Birmingham, Birmingham, UK; 4 Centre of Membrane Proteins and Receptors (COMPARE), Universities of Birmingham and Nottingham, Birmingham, UK; 5 Institute for Diabetes, Obesity & Metabolism and Department of Cell and Developmental Biology, Perelman School of Medicine, University of Pennsylvania, Philadelphia, PA USA; 6 Novo Nordisk Foundation Center for Basic Metabolic Research, University of Copenhagen, Copenhagen, Denmark; 7 Maine Medical Center Research Institute, Scarborough, ME USA; 8 Section on Integrative Physiology and Metabolism, Research Division, Joslin Diabetes Center, Harvard Medical School, Boston, MA USA; 9 Department of Orthopaedic Surgery, Beth Israel Deaconess Medical Center, Harvard Medical School, Boston, MA USA; 10 Department of Nutrition and Radiology, University of California, Davis, Davis, CA USA; 11 Department of Nutrition and Dietetics, Harokopio University of Athens, Athens, Greece; 12 Department of Kinesiology and Health, School of Arts and Sciences, Rutgers University, New Brunswick, NJ USA; 13 Division of Geriatrics and Nutritional Science, Washington University School of Medicine, St. Louis, MO USA; 14 Department of Physiology, The Johns Hopkins University School of Medicine, Baltimore, MD USA; 15 Diabetes, Endocrinology, and Obesity Branch, National Institute of Diabetes and Digestive and Kidney Diseases, National Institutes of Health, Bethesda, MD USA; 16 Steno Diabetes Center Aarhus, Aarhus University Hospital, Aarhus, Aarhus N, Denmark; 17 Department of Biomedicine, Aarhus University, Aarhus C, Denmark

**Keywords:** Biochemistry, Mechanisms of disease, Fat metabolism

## Abstract

Noradrenaline (NA) regulates cold-stimulated adipocyte thermogenesis^1^. Aside from cAMP signalling downstream of β-adrenergic receptor activation, how NA promotes thermogenic output is still not fully understood. Here, we show that coordinated α_1_-adrenergic receptor (AR) and β_3_-AR signalling induces the expression of thermogenic genes of the futile creatine cycle^2,3^, and that early B cell factors, oestrogen-related receptors and PGC1α are required for this response in vivo. NA triggers physical and functional coupling between the α_1_-AR subtype (ADRA1A) and Gα_q_ to promote adipocyte thermogenesis in a manner that is dependent on the effector proteins of the futile creatine cycle, creatine kinase B and tissue-non-specific alkaline phosphatase. Combined Gα_q_ and Gα_s_ signalling selectively in adipocytes promotes a continual rise in whole-body energy expenditure, and creatine kinase B is required for this effect. Thus, the ADRA1A–Gα_q_–futile creatine cycle axis is a key regulator of facultative and adaptive thermogenesis.

## Abstract (web summary)

Rahbani et al. show that the α_1_-adrenergic receptor potentiates thermogenesis in thermogenic adipocytes, acting via Gα_q_ signalling, creatine kinase B and tissue-non-specific alkaline phosphatase.

## Main

The release of NA from innervating sympathetic nerve terminals is understood to be the predominant physiological regulator of ligand-mediated adaptive and facultative adipocyte thermogenesis^4,5^. β-AR signalling and Gα_s_-coupled cAMP production have dominated the focus of sympathetic activation of adipocyte-mediated energy dissipation^6–8^. Although it has long been appreciated that NA engages G-protein-coupled receptors (GPCRs) aside from β-ARs on brown adipocytes^9^, an understanding of the signalling axes and effector pathways contributing to NA-stimulated adipocyte thermogenesis is still incomplete. Given the variability of existing brown adipose tissue (BAT) depots in humans, along with the reductions of BAT activity in obesity and ageing, defining the molecular mechanisms that promote the greatest activation of adipocyte thermogenesis is of considerable interest to uncover if BAT is a viable clinical target that can enhance cardiometabolic health^10,11^.

## Results

### The α-adrenergic receptor, encoded by *Adra1a*, is enriched in brown adipose tissue

**Fig. 1:**
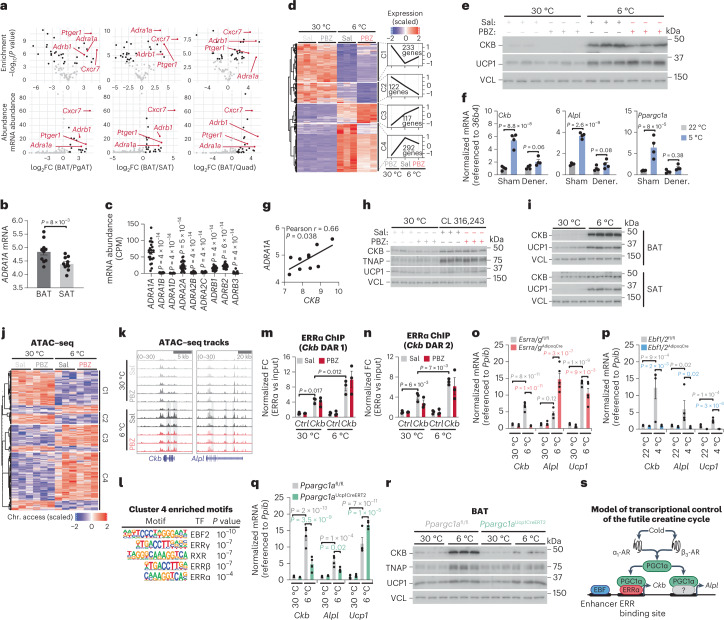
Regulation of CKB and TNAP expression. **a**, BAT-enriched (top; false discovery rate (FDR) < 0.0005) and BAT-abundant (bottom; 10% most abundant) GPCRs. log_2_FC, log2 fold change; PgAT, perigonadal adipose tissue; Quad, quadriceps. **b**, *ADRA1A* expression in human BAT (*n* = 10) and SAT (*n* = 10), first cohort. **c**, mRNA expression in human BAT (*n* = 23), second cohort. CPM, counts per million. **d**, Heat map of DEGs in BAT following 24 h of 6 °C exposure. **e**, Western blot of BAT from mice treated as in **d**. **f**, qPCR with reverse transcription (RT–qPCR) of sham or denervated BAT 24 h following 5 °C (*n* = 4 per group). **g**, Pearson correlation in human BAT, first cohort (*n* = 10). **h**, Western blot from BAT from wild-type (C57BL6/N) male mice, after 48 h of CL 316,243 (1 mg kg^−1^ body weight) or saline (*n* = 3 biologically independent samples). **i**, Western blot from BAT and SAT from wild-type male mice 48 h after 6 °C exposure (*n* = 4 per group)**. j**, Heat map showing ATAC–seq density of DARs proximal to DEGs from **d** (*n* = 3 per group). **k**, ATAC–seq tracks. Grey shading represents cold-stimulated DARs. **l**, Motifs of transcription factors enriched at DARs proximal to cluster 4 genes, and present at DARs proximal to both *Ckb* and *Alpl*. **m**,**n**, ChIP–qPCR of ERRα bound to *Ckb* DAR 1 (**m**) and DAR 2 (**n**) (*n* = 3 per group). **o**, RT–qPCR from BAT 24 h after 6 °C exposure (*n* = 5 per group, females). **p**, RT–qPCR from BAT following 7 d of 4 °C exposure (*n* = 3 for *Ebf1/2*^AdipoqCre^ at 4 °C; *n* = 4 for all other groups, males). **q**, RT–qPCR from BAT following 24 h of 6 °C exposure (*n* = 4 per group, males). **r**, Western blot of BAT harvested 48 h after 6 °C exposure (females; *n* = 3 per group). **s**, Model of transcriptional control of the futile creatine cycle. Data are presented as the mean ± s.e.m. and *n* indicates the number of biologically independent experiments. **b**,**f**, Two-tailed student’s *t*-tests; **c**, one-way analysis of variance (ANOVA; Tukey’s post-hoc test); **g**, Pearson correlation (two-sided); **m**–**q**, two-way ANOVA (Fisher’s least significant difference (LSD)). Source data

**Extended Data Fig. 1:**
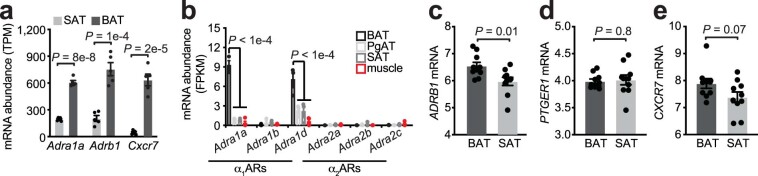
α-adrenergic receptor expression in murine and human adipose tissues. **a**, Ribosomal profiling of candidate GPCRs from SAT and BAT of 30 °C-acclimated mice^14^ (*n* = 5 per tissue). **b**, Ribosomal profiling from TRAP^13^ of α_1_AR and α_2_AR subtypes from different tissues in mice (BAT: *n* = 3, PgAT: *n* = 3, SAT: *n* = 3, QUAD muscle: *n* = 4). **c-e**, Relative mRNA expression of (**c**) *ADRB1*, (**d**) *PTGER1* and (**e**) *CXCR7* from human BAT (adipose tissue proximal to carotid sheath) (*n* = 10) and paired SAT (*n* = 10), first cohort. Data are presented as mean ± s.e.m. and *n* numbers are of biologically independent experiments. **a, c-e**, two-tailed student’s *t*-tests; **b**, one-way ANOVA (Fisher’s LSD). Source data

Because much of adipocyte thermogenesis is regulated by the sympathetic nervous system through GPCR signalling^6,7,12^, we analysed ribosomal profiling data^13^ to score GPCR mRNA expression in BAT based on mRNA enrichment and abundance. Four genes (*Adra1a*, *Adrb1*, *Ptger1* and *Cxcr7,* *also known as* *Ackr3*) fulfilled the criteria of being both BAT-enriched and abundant GPCRs (Fig. 1a). Analysis of an independent dataset^14^ confirmed brown adipocyte enrichment of *Adra1a*, *Adrb1* and *Cxcr7 (Ptger1* was not identified; Extended Data Fig. 1a). *Adra1a* was the most abundant α_1_-AR subtype in mouse brown adipocytes, followed by *Adra1d*, whereas *Adra1b* and all the α_2_-AR subtypes (encoded by *Adra2a*, *Adra2b* and *Adra2c*) were poorly expressed (Extended Data Fig. 1b). Of the GPCR candidates, *ADRA1A* was the most enriched in human deep BAT (proximal to the carotid sheath) over paired white subcutaneous adipose tissue (SAT; Fig. 1b), followed by *ADRB1* (Extended Data Fig. 1c), while *PTGER1* and *CXCR7* did not exhibit statistically significant BAT enrichment (Extended Data Fig. 1d,e). Analysis of RNA sequencing (RNA-seq) from an independent human cohort revealed that *ADRA1A* displayed the highest expression levels in supraclavicular adipose tissue (primary location of human BAT) compared to all α-AR and β-AR subtypes (Fig. 1c). These data prompted us to focus our attention on α-AR signalling in BAT.

### α_1_-adrenergic receptor signalling regulates *Ckb* and *Alpl* expression in the cold

**Extended Data Fig. 2:**
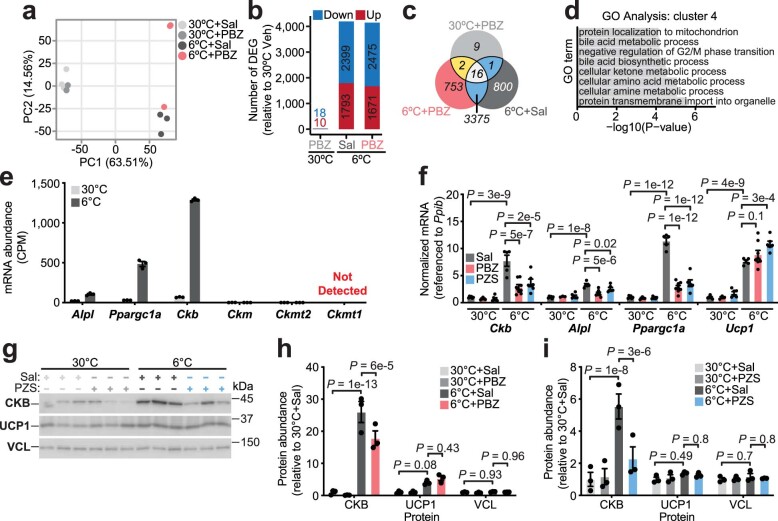
Regulation of the cold-stimulated BAT transcriptome by α-adrenergic receptor signalling. **a**, Principal component analysis (PCA) of gene expression from BAT transcriptomics of Fig. 1d. Percent of variance explained by each PC is shown. Phenoxybenzamine (PBZ) and Saline (Sal). **b**, Barplot showing the number of down-regulated and up-regulated genes, using “30 °C + Sal” condition as baseline from BAT transcriptomics of Fig. 1d. Differentially expressed genes (DEGs) are identified using cutoffs of (log_2_FC > 1 and FDR < 0.01). **c**, Venn diagram showing the overlap of DEGs between the 30 °C + PBZ (light gray), 6 °C + Sal (dark gray) and 6 °C + PBZ (red) from BAT transcriptomics of Fig. 1d. **d**, GO Term enrichment of Cluster 4 genes from BAT transcriptomics of Fig. 1d. **e**, RNA-seq of BAT transcriptomics of Fig. 1d. CPM (counts per million). **f**, RT-qPCR from BAT of wild-type male mice (C57BL6/N, 6–8 weeks of age), treated as in Fig. 1d. Prazosin (PZS) was injected (3 times over 24 hours) intraperitoneally (i.p.) at 5 mg kg^−1^ (30 °C: *n* = 6 Sal, *n* = 3 PBZ, *n* = 6 PZS) (6 °C: *n* = 5 Sal, *n* = 8 PBZ, *n* = 6 PZS). **g**, Western blot of BAT from wild-type male mice (C57BL6/N, 6–8 weeks of age), reared at 22 °C, housed at 30 °C for 5 days and then subjected to 30 °C or 6 °C at ZT4. 1 hour prior to onset of 6 °C exposure (ZT3), mice were injected i.p. with PZS (5 mg kg^−1^, injected 3 times over 24 hours) or Sal. BAT was harvested 24 hours following 6 °C exposure (*n* = 3 per group). **h-i**, Quantification of western blots from (**h**) Fig. 1e (*n* = 3 per group) and (**i**) Extended Data Fig. 2g (*n* = 3 per group). Data are presented as mean ± s.e.m. and *n* numbers are of biologically independent experiments. **f**, One-way ANOVA (Fisher’s LSD); **h, i**, Two-way ANOVA (Fisher’s LSD). Source data

**Extended Data Fig. 3:**
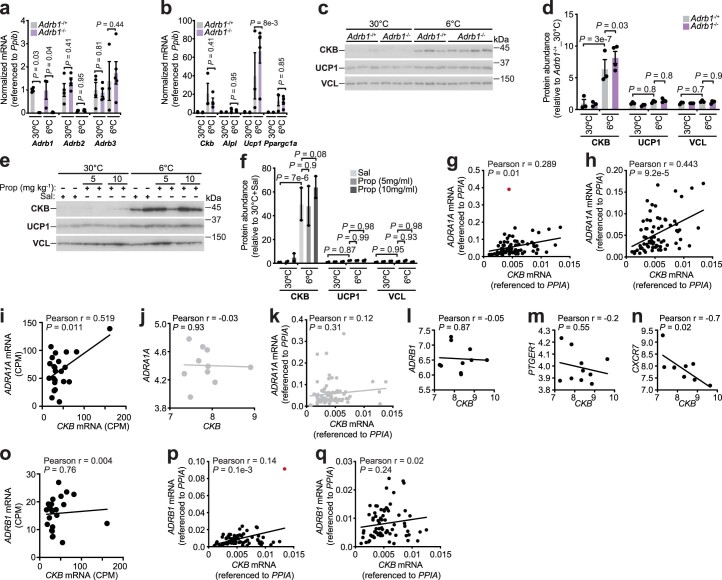
ADRA1A, not ADRB1, correlates with, CKB abundance in human BAT. **a-b**, RT-qPCR analysis of BAT from *Adrb1*^-/+^ and *Adrb1*^-/-^ female mice reared at 22 °C, housed at 30 °C for 5 days and then subjected to 30 °C or 6 °C at ZT4 for 24 hours (30 °C: *n* = 4 per genotype) (6 °C: *n* = 3 *Adrb1*^-/+^, *n* = 5 *Adrb1*^-/-^). **c**, Western blot from BAT of mice treated as in Extended Data Fig. 3a, b (30 °C: *n* = 3 per genotype) (6 °C: *n* = 3 *Adrb1*^-/+^, *n* = 4 *Adrb1*^-/-^). **d**, Quantification of western blots from Extended Data Fig. 3c (30 °C: *n* = 3 per genotype) (6 °C: *n* = 3 *Adrb1*^-/+^, *n* = 4 *Adrb1*^-/-^). **e**, Western blot from BAT of wild-type male mice (C57BL6/N, 6–8 weeks of age), reared at 22 °C, housed at 30 °C for 5 days and then subjected to 30 °C or 6 °C at ZT4. 1 hour prior to onset of 6 °C exposure (ZT3), mice were injected i.p. with propranolol hydrochloride (5 mg kg^−1^ or 10 mg kg^−1^) or saline (Sal). BAT was harvested 24 hours following 6 °C exposure (*n* = 2 per group). **f**, Quantification of western blots from Extended Data Fig. 3e (*n* = 2 per group). **g**, Pearson correlation of *CKB* with *ADRA1A* mRNA in human BAT (*n* = 73), third cohort. Red data point indicates outlier based on ROUT method (Q = 0.1%). **h**, Pearson correlation with outlier identified in Extended Data Fig. 3g removed. **i**, Pearson correlation of *CKB* with *ADRA1A* mRNA in human BAT (supraclavicular adipose tissue) (*n* = 23), second cohort. **j, k**, Pearson correlation of *CKB* with *ADRA1A* mRNA in (**j**) human SAT (superficial subcutaneous adipose tissue) (*n* = 10), first cohort and (**k**) human SAT (superficial neck adipose tissue) (*n* = 73), third cohort. **l-n**, Pearson correlation of *CKB* with (**l**) *ADRB1*, (**m**) *PTGER1* and (**n**) *CXCR7* mRNA in human BAT (adipose tissue proximal to carotid sheath) (*n* = 10), first cohort. **o**, Pearson correlation of *CKB* with *ADRB1* mRNA in human BAT (supraclavicular adipose tissue) (*n* = 23), second cohort. **p**, Pearson correlation of *CKB* with *ADRB1* mRNA in human BAT (*n* = 73), third cohort. Red data point indicates outlier based on ROUT method (Q = 0.1%). **q**, Pearson correlation with outlier identified in Extended Data Fig. 3p removed. Data are presented as mean ± s.e.m. and *n* numbers are of biologically independent experiments. **a, b, d, f**, Two-way ANOVA (Fisher’s LSD); **g-q**, Pearson correlation (two-sided). Source data

To explore the cold-stimulated transcriptional programme regulated by α-AR signalling, we first generated RNA-seq transcriptomes from BAT of mice pretreated with a single intraperitoneal (i.p.) injection of either the pan-α-AR antagonist phenoxybenzamine (PBZ) or saline control and then housed at 30 °C or 6 °C for 24 h. PBZ treatment essentially had no effect on gene expression at 30 °C (Extended Data Fig. 2a–c). We identified four gene clusters stratified by differential expression profiles (Fig. 1d and Supplementary Data 1). Cluster 4 was defined by cold-induced genes that were reduced in abundance by PBZ treatment (Fig. 1d). Analysis of this gene set revealed Gene Ontology (GO) term pathway enrichment of protein transmembrane import into organelle and protein localization to mitochondria, among others (Extended Data Fig. 2d). Notably, mRNAs encoding the effector proteins of the futile creatine cycle, creatine kinase B (*Ckb*, encoding CKB)^2^ and tissue-non-specific alkaline phosphatase (*Alpl*, encoding TNAP)^3^, were cold inducible in a α-AR-dependent manner (Supplementary Data 1). Similarly, peroxisome proliferator-activated receptor gamma co-activator 1 alpha (*Ppargc1a*) mRNA, encoding PGC1α, a co-activator of mitochondrial and thermogenic genes^15,16^, was a cold-stimulated PBZ target (Supplementary Data 1). As demonstrated previously in mouse adipose tissues (white, beige and brown)^2^, the other creatine kinase isoforms—*Ckm*, *Ckmt1* and *Ckmt2*—were poorly expressed or not even detected (Extended Data Fig. 2e). In striking contrast to the coordinated induction of *Ckb* and *Alpl* by cold exposure, no other creatine kinase isoform was cold inducible (Extended Data Fig. 2e). In a separate mouse cohort, both PBZ and a structurally distinct antagonist of α_1_-ARs, prazosin (PZS), inhibited the cold-stimulated induction of *Ckb*, *Alpl* and *Ppargc1a* mRNA in BAT (Extended Data Fig. 2f) and blunted the elevation of CKB protein in BAT (Fig. 1e and Extended Data Fig. 2g–i). In contrast, uncoupling protein 1 (encoded by *Ucp1*) mRNA and protein levels were unchanged by α-AR or α_1_-AR antagonism (Fig. 1e and Extended Data Fig. 2f–i). Next, we carried out unilateral denervation of the interscapular BAT (iBAT) depot in which the right lobe was surgically denervated while the left lobe remained intact^12^. The cold-mediated elevation of *Ckb*, *Alpl* and *Ppargc1a*—observed in the sham-operated lobe—was blocked in the sympathetically denervated BAT lobes (Fig. 1f), demonstrating that innervation of BAT by the sympathetic nervous system is required to elevate futile creatine cycling genes in response to cold. Even though *Adrb1* was BAT enriched (Extended Data Fig. 1a,c), ADRB1-dependent regulation of futile creatine cycling gene expression was ruled out because either genetic ablation or pharmacological inhibition of ADRB1 signalling did not block cold-stimulated *Ckb*, *Alpl* and *Ppargc1a* mRNA production or CKB protein induction in BAT (Extended Data Fig. 3a–f). Finally, *ADRA1A* was positively correlated with *CKB* in human BAT from three independent cohorts (Fig. 1g and Extended Data Fig. 3g–i). In contrast, *CKB* did not display a positive correlation with *ADRA1A* in human SAT (Extended Data Fig. 3j, k) nor a consistent association with *ADRB1*, *PTGER1* or *CXCR7* in human BAT (Extended Data Fig. 3l–q). Collectively, these data suggest that the cold-stimulated elevation of futile creatine cycling genes is regulated, at least in part, by the sympathetic nervous system through α_1_-AR signalling in BAT.

### CKB and TNAP expression through α_1_-AR and β_3_-AR signalling

**Extended Data Fig. 4:**
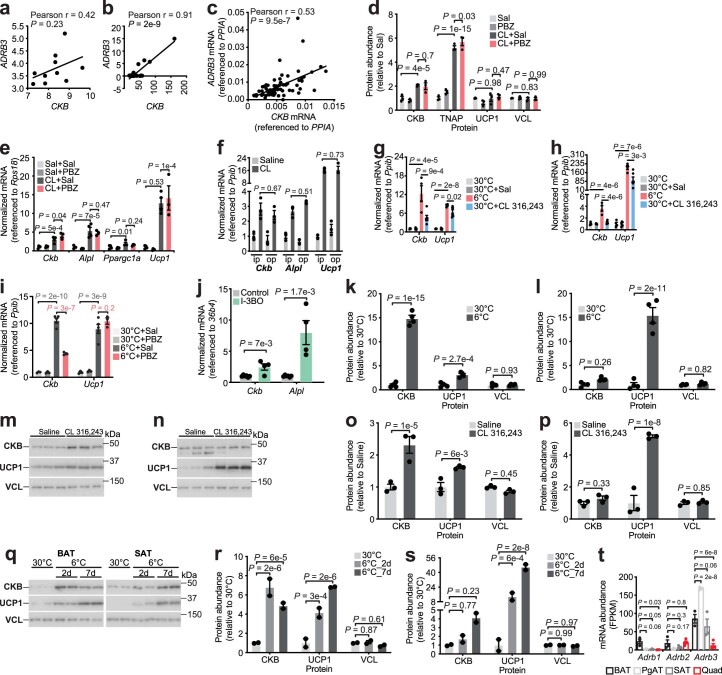
*Adrb3*, and Gα_s_ signaling, promote *Ckb* and *Alpl* expression. **a-c**, Pearson correlations of *CKB* with *ADRB3* mRNA in human BAT from the (**a**) first cohort (*n* = 10), (**b**) second cohort (*n* = 23), and (**c**) third cohort (*n* = 73). **d**, Western blot quantification from Fig. 1h (*n* = 3 per group). **e**, RT-qPCR of BAT from mice treated as in Fig. 1h (30 °C: *n* = 4 Sal, *n* = 4 PBZ) (6 °C: *n* = 4 Sal, *n* = 3 PBZ). **f**, intraperitoneal (ip) injections (5 injections over 48 hours) with CL 316,243 (1 mg kg^−1^) or Sal compared to osmotic pump (op) implantations above the interscapular BAT with CL 316,243 or Sal (*n* = 3 per group). **g-h**, RT-qPCR of (**g**) BAT and (**h**) SAT from wild-type male mice (C57BL6/N, 6–8 weeks of age), subjected to 30 °C or 6 °C (*n* = 4 per group) or injected i.p. (5 injections over 48 hours) with CL 316,243 or Sal at 30 °C (*n* = 6 per group). **i**, RT-qPCR analysis of BAT from wild-type male mice (C57BL6/N, 6–8 weeks of age), treated as in Fig. 1d (30 °C: *n* = 3 Sal, *n* = 3 PBZ) (6 °C: *n* = 5 Sal, *n* = 3 PBZ). **j**, RT-qPCR of BAT from I-3BO (*n* = 4) and control mice (*n* = 6). **k-l**, Western blot quantification from (**k**) BAT (*n* = 4 per group) and (**l**) SAT (*n* = 4 per group) from Fig. 1i. **m-n**, Western blot analysis of (**m**) BAT and (**n**) SAT from wild-type male mice (C57BL6/N, 6–8 weeks of age), reared at 22 °C, housed at 30 °C for 5 days and then injected i.p. (5 injections over 48 hours) with CL 316,243 or Sal (*n* = 3 per group). **o-p**, Western blot quantification from (**o**) Extended Data Fig. 4m (*n* = 3 per group) and (**p**) Extended Data Fig. 4n (*n* = 3 per group). **q**, Western blot from BAT and SAT of male wild-type mice (C57BL6/N, 6–8 weeks of age) subjected to 30 °C or 6 °C for 2 days (2d) or 7 days (7d) (*n* = 2 per group). **r-s**, Western blot quantification of Extended Data Fig. 4q from (**r**) BAT (*n* = 2 per group) and (**s**) SAT (*n* = 2 per group). **t**, Ribosomal profiling of βAR expression^13^ (BAT *n* = 3, SAT *n* = 3, PgAT *n* = 3, Quad muscle *n* = 4). Data are presented as mean ± s.e.m. and *n* numbers are of biologically independent experiments. **a-c**, Pearson correlation (two-sided)**; d, f, i, k, l, o, p, r, s, t**, Two-way ANOVA (Fisher’s LSD); **e, g, h**, One-way ANOVA (Fisher’s LSD); **j**, two-tailed student’s *t*-tests. Source data

Like *ADRA1A*, *ADRB3* exhibited a positive association with *CKB* in human BAT from three independent cohorts (Extended Data Fig. 4a–c). This was consistent with the capacity for pharmacological ADRB3 activation by CL 316,243 (CL) to increase CKB protein abundance in mouse BAT (Fig. 1h and Extended Data Fig. 4d). ADRB3 signalling also elevated TNAP protein abundance in mouse BAT (Fig. 1h and Extended Data Fig. 4d). Importantly, ADRB3-stimulated induction of *Ckb*, *Alpl*, *Ppargc1a* and *Ucp1* mRNA abundance and CKB, TNAP and UCP1 protein levels was not blocked by PBZ (Fig. 1h and Extended Data Fig. 4d,e), indicating that pan-α-AR blockade did not indirectly effect ADRB3-stimulated thermogenic gene induction. We did not detect any difference in the amount of *Ckb* (or *Alpl* and *Ucp1*) induction if CL was administered by twice daily i.p. injection or by continuous release through an osmotic pump (Extended Data Fig. 4f), suggesting that *Ckb* (and *Alpl* and *Ucp1*) induction by individual ADRB3 signalling occurs similarly whether stimulated transiently or continuously. Nevertheless, the cold-stimulated induction of *Ckb* in BAT was higher (about 12-fold) than the elevation of *Ckb* through ADRB3 agonism (about 4.5-fold), whereas the induction of *Ucp1* mRNA expression by these interventions was comparable (Extended Data Fig. 4g). A similar preferential induction of *Ckb* by cold (about 3.6-fold) over ADRB3 agonism (about 1.2-fold) was displayed in SAT (Extended Data Fig. 4h). Finally, *Ckb* levels in BAT following ADRB3 activation (Extended Data Fig. 4g) mirrored the remaining levels of *Ckb* in α-AR-inhibited cold-activated BAT (Extended Data Figs. 2f and 4i), suggesting that the residual cold-stimulated induction of *Ckb* during α-AR or α_1_-AR blockade was mediated by the Gα_s_–cAMP signalling axis downstream of ADRB3 activation. Consistent with the idea that Gα_s_–cAMP signalling promotes futile creatine cycling gene expression, inducible overexpression of G-protein-coupled receptor 3 (GPR3, a ligand-independent regulator of the Gα_s_–cAMP pathway)^12^ in *Ucp1*^+^ cells was sufficient to enhance *Ckb* and *Alpl* mRNA abundance (Extended Data Fig. 4j). Cold exposure or ADRB3 agonism both elicited a greater relative induction of *Ckb* mRNA and protein in BAT compared to SAT (Fig. 1i and Extended Data Fig. 4k–s), even though *Adrb3* expression was comparable between these tissues (Extended Data Fig. 4t)^13^. These data indicate that, in addition to BAT-selective α_1_-AR signalling, intracellular factors contribute towards the priming of brown adipocytes to trigger CKB expression downstream of Gα_s_ signalling. It is noteworthy that CKB protein could be induced in SAT following 1 week of cold exposure (Extended Data Fig. 4q,s), suggesting that its expression was commensurate with beige adipogenesis. Collectively, our data imply that the Gα_s_–cAMP pathway promotes futile creatine cycling gene expression in thermogenic adipocytes and that this process is potentiated by α_1_-AR signalling.

### Transcriptional regulators of the futile creatine cycle

**Extended Data Fig. 5:**
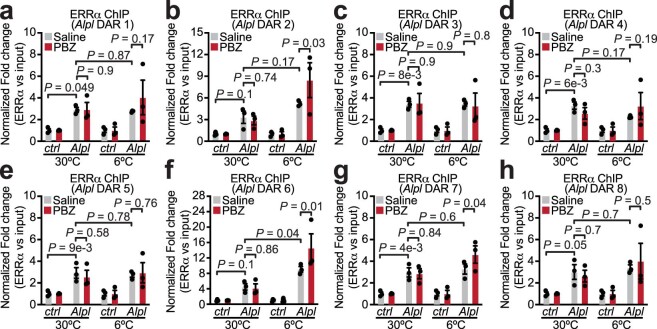
ChIP-qPCR of ERRα bound to cold-responsive DARs near *Alpl*. **a-h**, Chromatin immunoprecipitation coupled to qPCR (ChIP-qPCR) of ERRα bound to *Alpl* (**a**) DAR 1, (**b**) DAR 2, (**c**) DAR 3, (**d**) DAR 4, (**e**) DAR 5, (**f**) DAR 6, (**g**) DAR 7, and (**h**) DAR 8. Wild-type male mice (C57BL6/N, 6–8 weeks of age) were reared at 22 °C, housed at 30 °C for 5 days and then subjected to 30 °C or 6 °C at ZT4. 1 hour prior to onset of 6 °C exposure (ZT3), mice were injected intraperitoneally (i.p.) with PBZ (5 mg kg^−1^) or Saline (*n* = 3 per group). Chromatin was prepared for ERRα ChIP from freshly isolated BAT 24 hours after onset of 6 °C exposure. Data are presented as mean ± s.e.m. and *n* numbers are of biologically independent experiments. **a-h**, Two-way ANOVA (Fisher’s LSD). Source data

Using assay for transposase-accessible chromatin sequencing (ATAC–seq) of BAT nuclei, we identified differentially accessible regions (DARs) proximal to the differentially expressed genes (DEGs) of our BAT transcriptomes (Fig. 1j). We next identified transcription factor motifs that were: (1) statistically enriched in DARs proximal to cluster 4 genes and (2) present in cold-stimulated DARs proximal to both *Ckb* and *Alpl* (Fig. 1k). We found oestrogen-related receptor (ERR) and early B cell factor (EBF) response elements to be most enriched (Fig. 1l). ERRα and its co-activator PGC1α are known transcriptional regulators of the cold response, and EBF2 facilitates their binding on target thermogenic genes^16^. We explored the chromatin occupancy of ERRα at the cold-responsive DARs proximal to *Ckb* and *Alpl* in BAT using chromatin immunoprecipitation coupled to quantitative PCR (ChIP–qPCR). At 30 °C, ERRα binding to DARs proximal to both *Ckb* and *Alpl* was enriched (by about fourfold) over a control region that is not bound by ERRα (Fig. 1m,n and Extended Data Fig. 5). Around 6 °C of exposure further enhanced the occupancy of ERRα (by twofold over 30 °C) on all cold-responsive DARs proximal to *Ckb* (Fig. 1m,n), but not to those proximal to *Alpl* (Extended Data Fig. 5). Inhibition of α-AR signalling with PBZ did not alter chromatin accessibility (Fig. 1j,k) or ERRα occupancy on DARs proximal to *Ckb* and *Alpl* (Fig. 1m,n and Extended Data Fig. 5). Thus, antagonism of α-AR signalling during cold exposure does not influence chromatin binding activity, but may regulate futile creatine cycling gene expression through transcription factor co-activation or mRNA stability.

**Extended Data Fig. 6:**
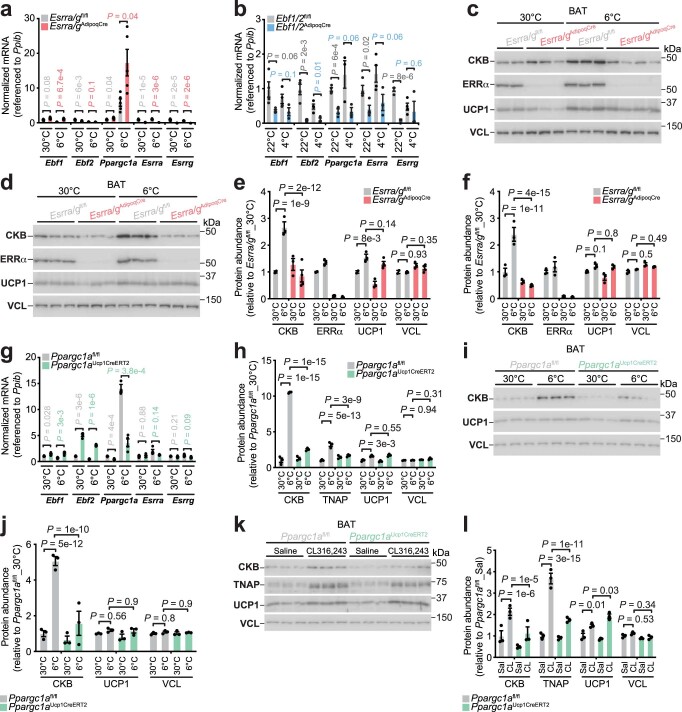
Transcriptional control of cold-stimulated futile creatine cycling gene expression. **a-b**, RT-qPCR from BAT of male (**a**) *Esrra*/*g*^AdipoqCre^ and *Esrra*/*g*^fl/fl^ mice (6 °C for 24 hours) (*n* = 5 per group) and (**b**) *Ebf1/2*^AdipoqCre^ and *Ebf1/2*^fl/fl^ mice (4 °C for 7 days) (*n* = 3 for *Ebf1/2*^AdipoqCre^ at 4 °C; *n* = 4 for all other groups). **c-d**, Western blot analysis of BAT from (**c**) male (*Esrra/g*^fl/fl^: *n* = 2 for 30 °C, *n* = 3 for 6 °C) *(Esrra*/*g*^AdipoqCre^: *n* = 3 for 30 °C, *n* = 4 for 6 °C) and (**d**) female mice (*n* = 3 per group) subjected to 30 °C or 6 °C for 24 hours. **e-f**, Western blot quantification from (**e**) Extended Data Fig. 6c (*Esrra/g*^fl/fl^: *n* = 2 for 30 °C, *n* = 3 for 6 °C) *(Esrra*/*g*^AdipoqCre^: *n* = 3 for 30 °C, *n* = 4 for 6 °C) and (**f**) Extended Data Fig. 6d (*n* = 3 per group). **g**, RT-qPCR from BAT of *Ppargc1a*^Ucp1CreERT2^ and *Ppargc1a*^fl/fl^ mice, reared at 22 °C, housed at 30 °C for 5 days and then subjected to 30 °C or 6 °C for 24 hours (*n* = 4 per group). **h**, Western blot quantification from Fig. 1r (*n* = 3 per group). **i**, Western blot of BAT from male mice (6–8 weeks of age), treated as in Extended Data Fig. 6g. **j**, Western blot quantification from Extended Data Fig. 6i (*n* = 3 per group). **k**, Western blot analysis of BAT from female mice (6–8 weeks of age), reared at 22 °C, housed at 30 °C for 5 days and then injected i.p. (5 injections over 48 hours) with CL 316,243 (1 mg kg^−1^) or saline at 30 °C (*n* = 3 per group). **l**, Western blot quantification from Extended Data Fig. 6k (*n* = 3 per group). Data are presented as mean ± s.e.m. and *n* numbers are of biologically independent experiments. **a, b, g**, multiple two-tailed student’s *t*-tests (Holm-Šidák test); **e, f, h, j, l**, Two-way ANOVA (Fisher’s LSD). Source data

Next, we sought to determine if ERR, EBF and PGC1α regulate futile creatine cycling gene expression in BAT in vivo. ERRγ (encoded by *Esrrg*) can compensate for loss of ERRα (encoded by *Esrra*)^17^ and *Ebf1* can compensate for *Ebf2* deletion^16^. Adipocyte-selective co-deletion of either *Esrra*/*Esrrg* (*Esrra*/*g*^AdipoqCre^; Extended Data Fig. 6a) or *Ebf1/Ebf2* (*Ebf1/2*^AdipoqCre^; Extended Data Fig. 6b) completely blocked the cold-induced increase of *Ckb* mRNA in BAT (Fig. 1o,p). The cold-stimulated induction of CKB protein was also fully reliant on *Esrra*/*Esrrg* (Extended Data Fig. 6c–f). On the other hand, while the induction of *Alpl* mRNA by cold was reliant on *Ebf1* and *Ebf2* (Fig. 1p), *Esrra* and *Esrrg* were dispensable (Fig. 1o), fully consistent with our ChIP–qPCR analysis (Extended Data Fig. 5). Surprisingly, the elevation of *Ucp1* mRNA and protein by cold was largely independent of *Esrra*/*Esrrg* (Fig. 1o and Extended Data Fig. 6c–f). We discovered that *Alpl* and *Ppargc1a* were both induced to a higher level in BAT of cold-exposed *Esrra*/*g*^AdipoqCre^ mice compared to control mice (Fig. 1o and Extended Data Fig. 6a). Thus, because *Alpl* and *Ppargc1a* mirrored one another after cold exposure in *Esrra*/*g*^AdipoqCre^ mice, and because *Alpl*, *Ppargc1a* and *Ckb* mRNA levels were all coordinately dependent following cold-stimulated α_1_-AR signalling (Extended Data Fig. 2f), we hypothesized that the cold-stimulated expression of futile creatine cycling genes are synchronized through PGC1α. Thus, we constructed mice with inducible deletion of *Ppargc1a* in *Ucp1*^*+*^ cells (*Ppargc1a*^Ucp1CreERT2^). Tamoxifen-mediated reduction of *Ppargc1a* in this model exhibited a similar level of diminution as with pharmacological α-AR antagonism, while *Ebf1*, *Ebf2*, *Esrra* and *Esrrg* mRNA levels were not reduced, and even upregulated (Extended Data Fig. 6g). Strikingly, genetic depletion of *Ppargc1a* diminished the cold-stimulated induction of both *Ckb* (by about 65%) and *Alpl* (by about 45%) mRNA (Fig. 1q) and blocked the cold-mediated elevation of CKB and TNAP protein (Fig. 1r and Extended Data Fig. 6h–j). Similarly, the ADRB3-stimulated induction of both CKB and TNAP was reduced in BAT of *Ppargc1a*^Ucp1CreERT2^ mice compared to *Ppargc1a*^fl/fl^ mice (Extended Data Fig. 6k,l). In contrast, *Ppargc1a* was dispensable for the induction of *Ucp1* mRNA and protein by cold exposure and ADRB3 agonism (Fig. 1q,r and Extended Data Fig. 6h–l). Together, our findings show that the expression of both *Ckb* and *Alpl* is dependent on *Ebf1*/*Ebf2* and *Ppargc1a* in brown adipocytes in response to cold. However, while elevation of *Ckb* expression by cold depends on *Esrra*/*Esrrg*, cold-stimulated *Alpl* abundance is independent of *Esrra*/*Esrrg* and transcriptionally regulated by factors that remain to be defined. Our data are consistent with a model wherein PGC1α abundance is regulated by environmental cold through combined α_1_-AR and β_3_-AR signalling (other factors that trigger Gα_s_ signalling, such as GPR3, likely also play a role) to control futile creatine cycling gene expression in brown adipocytes (Fig. 1s).

### ADRA1A, CKB and TNAP mediate thermogenesis by noradrenaline

**Fig. 2:**
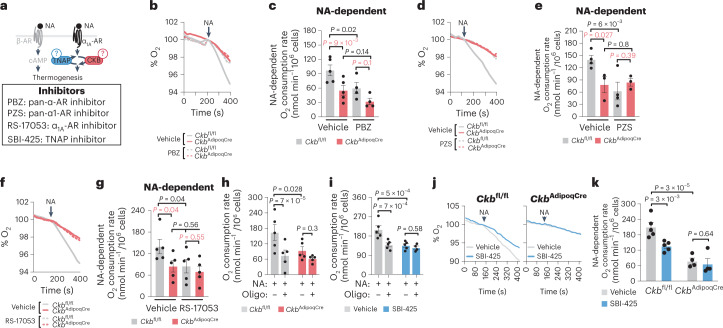
Noradrenaline-stimulated thermogenesis requires ADRA1A signalling, CKB and TNAP. **a**, Cartoon of approach to determine if α-AR, α_1_-AR and α_1A_-AR signalling as well as if CKB and TNAP are necessary for NA-stimulated brown adipocyte thermogenesis. **b**,**d**,**f**,**j**, Representative basal and NA-stimulated (0.1 μM) oxygen consumption traces of freshly isolated *Ckb*^fl/fl^ and *Ckb*^AdipoqCre^ brown adipocytes, treated with PBZ (1 μM) (**b**), PZS (1 μM) (**d**), RS-17053 (10 μM) (**f**) or SBI-425 (10 μM) (**j**), each compared to vehicle control. The time of NA addition (arrow) was normalized to 100% for ease of viewing the representative traces. **c**,**e**,**g**,**k**, NA-stimulated oxygen consumption rates (above basal) of freshly isolated *Ckb*^fl/fl^ and *Ckb*^AdipoqCre^ brown adipocytes, treated with PBZ (*n* = 5 per group) (**c**), PZS (*n* = 4 for *Ckb*^fl/fl^; *n* = 3 for *Ckb*^AdipoqCre^) (**e**), RS-17053 (*n* = 5 per group) (**g**) or SBI-425 (*n* = 5 SBI-425 and vehicle for *Ckb*^fl/fl^; *n* = 4 SBI-425 and vehicle for *Ckb*^AdipoqCre^) (**k**) each compared to vehicle control. In **k**, the NA-stimulated rates of *Ckb*^fl/fl^ brown adipocytes (vehicle and SBI-425) are the same as in **i**, and are shown for comparison to the *Ckb*^AdipoqCre^ brown adipocytes. **h**,**i**, ATP synthase-dependent NA-stimulated oxygen consumption rates (above basal) of freshly isolated *Ckb*^fl/fl^ (*n* = 5 per group) (**h**) and *Ckb*^AdipoqCre^ (*n* = 5 per group) brown adipocytes or *Ckb*^fl/fl^ brown adipocytes treated with SBI-425 (*n* = 5 for NA; *n* = 4 for NA + oligomycin) or vehicle (*n* = 5 per group) (**i**). Data are presented as the mean ± s.e.m. and *n* indicates the number of biologically independent experiments. **c**,**e**,**g**,**h**,**i**,**k**, Two-way ANOVA (Fisher’s LSD). Source data

**Extended Data Fig. 7:**
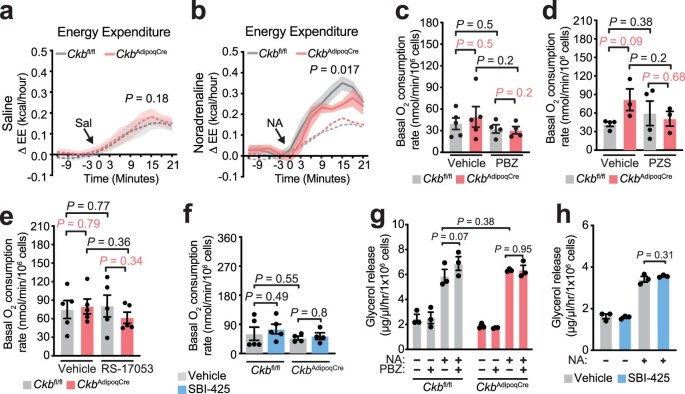
Basal respiration and lipolysis are not effected by inhibition of CKB, TNAP or αAR signaling. **a-b**, Energy expenditure (EE) of *Ckb*^fl/fl^ and *Ckb*^AdipoqCre^ male mice (n = 8 per group) reared at 22 °C and then housed for 5 days at 30 °C prior to administration of (**a**) saline (Sal) or (**b**) noradrenaline (NA, 1 mg kg^−1^). **c-f**, Basal oxygen consumption rates (related to experiments in Fig. 2b–g, j, k) of freshly isolated *Ckb*^fl/fl^ and *Ckb*^AdipoqCre^ brown adipocytes, treated with (**c**) PBZ (*n* = 5 per group), (**d**) PZS (*n* = 4 *Ckb*^fl/fl^; *n* = 3 *Ckb*^AdipoqCre^), (**e**) RS-17053 (*n* = 5 per group), or (**f**) SBI-425 (*Ckb*^fl/fl^: *n* = 5 per group; *Ckb*^AdipoqCre^: *n* = 4 per group). **g-h**, Glycerol release from freshly isolated (**g**) *Ckb*^fl/fl^ and *Ckb*^AdipoqCre^ brown adipocytes treated with PBZ compared to vehicle (*n* = 3 per group) or (**h**) brown adipocytes treated with SBI-425 compared to vehicle (*n* = 3 per group). Data are presented as mean ± s.e.m. and *n* numbers are of biologically independent experiments. **a, b**, Two-way ANOVA (Fisher’s LSD) from minutes 0 to 21; **c-g**, Two-way ANOVA (Fisher’s LSD); **h**, One-way ANOVA (Fisher’s LSD). Source data

GPCR signalling regulates both the acute activation of thermogenesis and the reconfiguration of transcriptional networks to support enhanced catabolic demand^8,12,18^. Because our data suggested that cold-stimulated α_1_-AR stimulation promotes *Ckb* and *Alpl* expression, we explored the possibility that facultative thermogenesis downstream of NA-stimulated α_1_-AR signalling is mediated through the futile creatine cycle. First, we injected mice with NA, which stimulated an increase in whole-body energy expenditure above the stress response elicited by saline injections. This response was decreased in fat-selective *Ckb* knockout (*Ckb*^AdipoqCre^) mice compared to control *Ckb*^fl/fl^ mice (Extended Data Fig. 7a,b). These data are consistent with the recently established role for CKB in thermogenesis by the futile creatine cycle^2^. However, given that in vivo administration of NA does not confine adrenergic signalling to fat, we sought to delineate the adipocyte-intrinsic regulation of NA-stimulated respiration by the futile creatine cycle through α_1_-AR signalling (Fig. 2a). Importantly, the NA-stimulated rise in respiration of acutely isolated *Ckb*^AdipoqCre^ brown adipocytes was substantially impaired (by about 45%) compared to *Ckb*^fl/fl^ brown adipocytes (Fig. 2b–g). Moreover, inhibition of α-AR, α_1_-AR or α_1A_-AR signalling blunted NA-stimulated respiration of *Ckb*^fl/fl^ control, but not *Ckb*^AdipoqCre^, brown adipocytes (Fig. 2b–g). Impeding mitochondrial ATP synthesis reduced NA-stimulated respiration of *Ckb*^fl/fl^ control brown adipocytes to a similar extent as *Ckb* deficiency, but had no effect on NA-stimulated respiration of *Ckb*^AdipoqCre^ brown adipocytes (Fig. 2h). Similarly, inhibition of TNAP activity decreased NA-mediated respiration in an ATP synthase-dependent manner (Fig. 2i). Furthermore, inhibition of TNAP activity decreased NA-stimulated respiration of *Ckb*^fl/fl^ control, but not *Ckb*^AdipoqCre^ brown adipocytes (Fig. 2j,k). In contrast, CKB deficiency, TNAP blockade or inhibition of α-AR, α_1_-AR or α_1A_-AR signalling had no effect on unstimulated (basal) respiration (Extended Data Fig. 7c–f). CKB deficiency, TNAP inhibition or α-AR antagonism had no effect on NA-stimulated lipolysis (Extended Data Fig. 7g,h), indicating that the diminished capacity to promote adrenergic-stimulated thermogenesis following reduction of these mechanisms was not a result of impaired substrate mobilization. Together, these data indicate that CKB, TNAP and ADRA1A signalling regulate NA-mediated respiration by promoting ATP turnover, affirming the link between NA-stimulated thermogenesis and the futile creatine cycle.

**Fig. 3:**
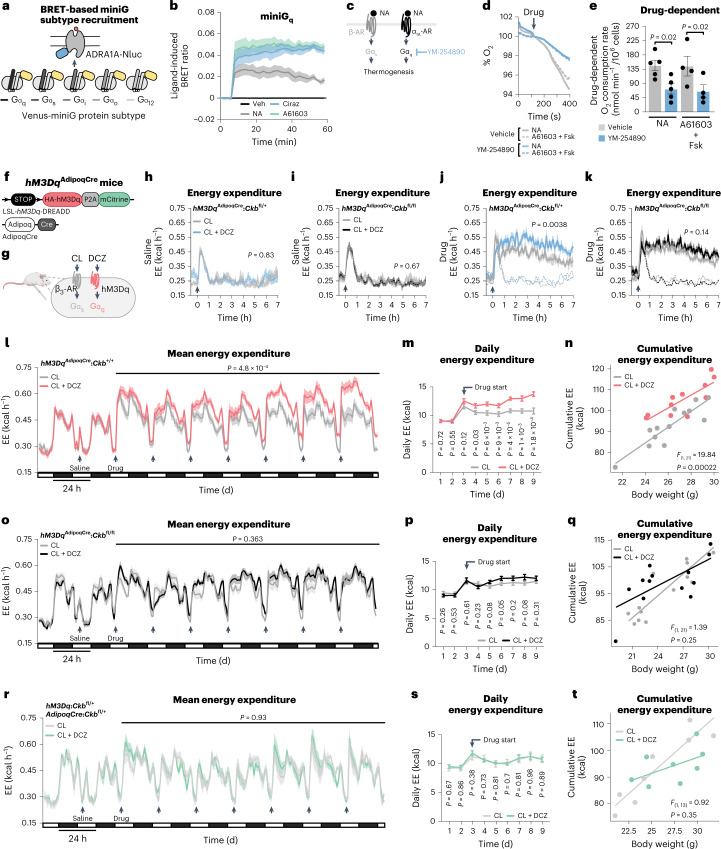
Thermogenesis by combined Gα_q_ and Gα_s_ signalling genetically requires *Ckb* in vivo. **a**, Schematic of BRET assay. **b**, Agonist-induced BRET in immortalized brown adipocytes (*n* = 3 per group). **c**, Cartoon depicting inhibition of Gα_q_ signalling by YM-254890. **d**, Representative basal and NA-stimulated (0.1 μM) oxygen consumption trace of freshly isolated brown adipocytes, treated with YM-254890 (10 μM) or vehicle. The time of NA addition (arrow) was normalized to 100%. **e**, NA-stimulated oxygen consumption rates (above basal) of freshly isolated brown adipocytes (*n* = 5 per group). **f**, Cartoon of *hM3Dq*^AdipoqCre^ mouse construction. **g**, Schematic of activation of Gα_s_ and Gα_q_ signalling in adipocytes from *hM3Dq*^AdipoqCre^ mice. **h**,**i**, Energy expenditure (EE) with saline injection of *hM3Dq*^AdipoqCre^:*Ckb*^fl/+^ mice (CL group, *n* = 6 males, 4 females; CL + DCZ group, *n* = 8 males, 4 females) (**h**) or *hM3Dq*^AdipoqCre^:*Ckb*^fl/fl^ mice (CL group, *n* = 11 males, 7 females; CL + DCZ group, *n* = 10 males, 7 females) (**i**). **j**,**k**, EE with drug injection of *hM3Dq*^AdipoqCre^:*Ckb*^fl/+^ mice (CL, *n* = 6 males, 4 females; CL + DCZ, *n* = 8 males, 4 females) (**j**) or *hM3Dq*^AdipoqCre^:*Ckb*^fl/fl^ mice (CL, *n* = 11 males, 7 females; CL + DCZ, *n* = 10 males, 7 females) (**k**). **l**–**n**, Mean EE (**l**), daily EE (**m**) and cumulative EE (**n**) of *hM3Dq*^AdipoqCre^:*Ckb*^+/+^ mice (CL, *n* = 8 males, 5 females; CL + DCZ, *n* = 8 males, 3 females). **o**–**q**, Mean EE (**o**), daily EE (**p**) and cumulative EE (**q**) of *hM3Dq*^AdipoqCre^:*Ckb*^fl/fl^ mice (CL, *n* = 7 males, 7 females; CL + DCZ, *n* = 7 males, 7 females). **r**–**t**, Mean EE (**r**), daily EE (**s**) and cumulative EE (**t**) of control (*hM3Dq*:*Ckb*^fl/+^ or *AdipoqCre*:*Ckb*^fl/+^) mice (*n* = 4 males, 4 females). Data are presented as the mean ± s.e.m. and *n* indicates the number of biologically independent experiments. **e**, two-tailed student’s *t*-test; **h**–**m**,**o**,**p**,**r**,**s**, two-way ANOVA (Fisher’s LSD); **n**,**q**,**t**, analysis of covariance (ANCOVA; two-sided, Bonferroni). Source data

**Extended Data Fig. 8:**
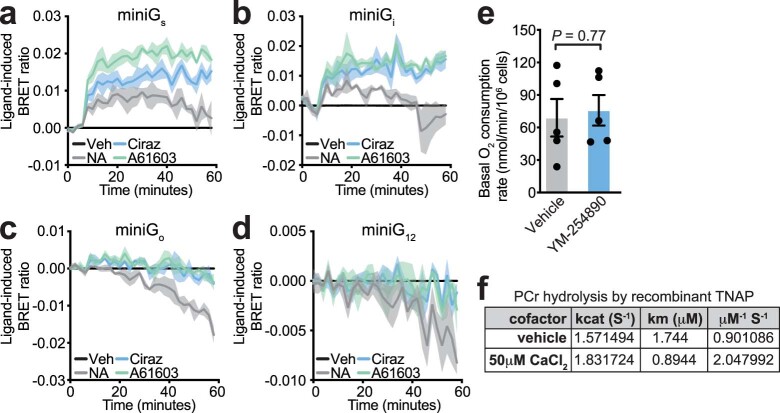
Physical and functional coupling of ADRA1A to Gα_q_^.^ **a-d**, agonist-induced BRET between ADRA1A-tagged Nano Luciferase (ADRA1A-Nluc) and Venus-tagged (**a**) miniGα_s_, (**b**) miniGα_i_, (**c**) miniGα_o_, and (**d**) miniGα_12_ protein sensors in immortalized brown adipocytes (*n* = 3 per group). **e**, Basal oxygen consumption rates (related to experiments in Fig. 3d, e) of freshly isolated brown adipocytes treated with YM-254890 compared to vehicle (*n* = 5 per group). **f**, Catalytic properties of recombinant TNAP (*n* = 3 per group). Data are presented as mean ± s.e.m. and *n* numbers are of biologically independent experiments. **e**, two-tailed student’s *t*-test. Source data

The G-protein class that couples ADRA1A activation to signalling in the interior of brown adipocytes has never been defined. Thus, we examined the G-protein-coupling profile of ADRA1A by measuring agonist-induced bioluminescence resonance energy transfer (BRET) between ADRA1A-tagged Nano Luciferase (ADRA1A-Nluc) and Venus-tagged miniG protein probes in immortalized brown adipocytes (Fig. 3a). NA, or selective agonism of α_1A_-AR (A61603) or α_1_-AR (cirazoline) signalling caused rapid physical coupling of ADRA1A-Nluc primarily to Gα_q_-Venus (Fig. 3b and Extended Data Fig. 8a–d), leading us to hypothesize that Gα_q_ signalling is functionally connected to ADRA1A following its engagement by NA (Fig. 3c). Consistent with this, YM-254890 (a selective Gα_q_ inhibitor)^19^ decreased NA-stimulated respiration (Fig. 3d,e) without any effect on basal respiration (Extended Data Fig. 8e). Activation of α_1_-ARs present on mature brown adipocytes elevates the production of inositol 1,4,5-triphosphate to trigger the release of calcium from intracellular stores^20,21^. Moreover, hydrolysis of the reference substrate p-nitrophenyl phosphate by TNAP is promoted by calcium^22^. However, whether calcium can stimulate TNAP-mediated hydrolysis of phosphocreatine is unknown. Strikingly, calcium, at physiologically relevant levels^23^, doubled the catalytic efficiency of recombinant TNAP (Extended Data Fig. 8f). Our data imply that the ADRA1A–Gα_q_ signalling axis promotes the elevation of calcium to control TNAP activity. This is in agreement with the regulation of TNAP activity downstream of α-AR engagement by NA in other biological systems (such as mesenteric vasculature)^24^. Together, our data suggest that stimulation of TNAP activity by calcium, at least partly, links ADRA1A-mediated Gα_q_ signalling to thermogenesis by the futile creatine cycle.

### Gα_q_ signalling potentiates thermogenesis through CKB in vivo

**Extended Data Fig. 9:**
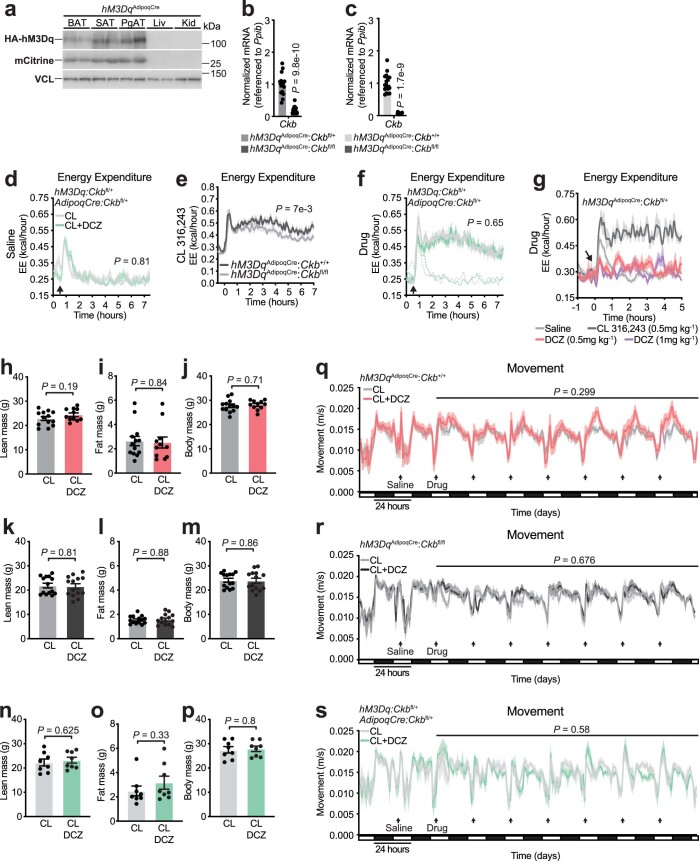
Energy expenditure by Gα_s_ and Gα_q_ signaling *in vivo*. **a**, Western blot of BAT, SAT, PgAT, liver (Liv) and kidney (Kid) (*n* = 2 per group). **b**, RT-qPCR from BAT (*hM3Dq*^AdipoqCre^:*Ckb*^fl/+^: *n* = 17, 9 males, 8 females) (*hM3Dq*^AdipoqCre^:*Ckb*^fl/fl^: *n* = 14, 6 males, 8 females). **c**, RT-qPCR from BAT (*n* = 16: 8 males, 8 females per genotype). **d**, EE of *hM3Dq*:*Ckb*^fl/+^ or *AdipoqCre*:*Ckb*^fl/+^ mice injected i.p. with saline (*n* = 12: 8 males, 4 females for both groups). **e**, EE of *hM3Dq*^AdipoqCre^:*Ckb*^+/+^ (*n* = 14: 8 males, 6 females) and *hM3Dq*^AdipoqCre^:*Ckb*^fl/fl^ (*n* = 16: 8 males, 8 females) following i.p. injection of CL (0.5 mg kg^−1^). **f**, EE of *hM3Dq*:*Ckb*^fl/+^ or *AdipoqCre*:*Ckb*^fl/+^ mice injected i.p. with CL or CL + DCZ (*n* = 12: 8 males, 4 females for both groups). **g**, EE of *hM3Dq*^AdipoqCre^:*Ckb*^fl/+^ male mice in response to saline (*n* = 16), CL (*n* = 8), DCZ (0.5 mg kg^−1^) (*n* = 8), or DCZ (1 mg kg^−1^) (*n* = 8). **h-j**, Baseline (**h**) lean mass, (**i**) fat mass, and (**j**) body mass of *hM3Dq*^AdipoqCre^:*Ckb*^+/+^ mice (CL: *n* = 13: 8 males, 5 females; CL + DCZ: *n* = 12: 8 males, 3 females). **k-m**, Baseline (**k**) lean mass, (**l**) fat mass, and (**m**) body mass of *hM3Dq*^AdipoqCre^:*Ckb*^fl/fl^ mice (CL: *n* = 14: 7 males, 7 females; CL + DCZ: *n* = 14: 7 males, 7 females). **n-p**, Baseline (**n**) lean mass, (**o**) fat mass, and (**p**) body mass of *hM3Dq*:*Ckb*^fl/+^ or *AdipoqCre*:*Ckb*^fl/+^ mice (*n* = 8 per group: 4 males, 4 females). **q-s**, movement of (**q**) *hM3Dq*^AdipoqCre^:*Ckb*^+/+^ mice (CL: *n* = 13: 8 males, 5 females; CL + DCZ: *n* = 11: 8 males, 3 females), (**r**) *hM3Dq*^AdipoqCre^:*Ckb*^fl/fl^ mice (CL: *n* = 14: 7 males, 7 females; CL + DCZ: *n* = 14: 7 males, 7 females), and (**s**) control (*hM3Dq*:*Ckb*^fl/+^ or *AdipoqCre*:*Ckb*^fl/+^) mice (*n* = 8 per group: 4 males, 4 females). Data are presented as mean ± s.e.m. and *n* numbers are of biologically independent experiments. **b, c, h-p** two-tailed student’s *t*-test; **d-f, q-s**, Two-way ANOVA (Fisher’s LSD). Source data

To determine if Gα_q_ signalling in adipocytes regulates energy expenditure in vivo, we used designer receptors exclusively activated by designer drugs (DREADD)-based chemogenetics^25^. We generated mice that conditionally express a haemagglutinin (HA)-tagged modified muscarinic receptor (HA-hM3Dq) selectively in adipocytes (*hM3Dq*^AdipoqCre^; Fig. 3f and Extended Data Fig. 9a). Fat-selective hM3Dq expression renders these mice capable of activating Gα_q_ signalling in adipocytes following deschloroclozapine (DCZ) administration^26^ (Fig. 3g). Because mouse adipocytes (brown, beige or white) primarily express *Ckb* (the other isoenzymes encoded by *Ckmt1*, *Ckmt2* and *Ckm* are expressed at low to non-existent levels)^2^ (Extended Data Fig. 2e), and because CKB is responsible for the bulk of creatine kinase activity within mouse thermogenic brown adipocytes^2^, we mated *hM3Dq*^AdipoqCre^ mice to *Ckb* conditional mice to construct mice where Gα_q_ signalling could be activated selectively in *Ckb*-expressing (*hM3Dq*^AdipoqCre^:*Ckb*^+/+^ and *hM3Dq*^AdipoqCre^:*Ckb*^fl/+^) or *Ckb-*deficient (*hM3Dq*^AdipoqCre^:*Ckb*^fl/fl^) adipocytes (Extended Data Fig. 9b,c). Mice were single housed at 30 °C and injected i.p. with saline. This caused a transient spike in energy expenditure that subsided rapidly within 1 h (Fig. 3h,i and Extended Data Fig. 9d). Next, we triggered either Gα_s_ signalling alone (by activating ADRB3 with CL) or both Gα_s_ and Gα_q_ signalling (through combined ADRB3 and hM3Dq stimulation with CL and DCZ, respectively; Fig. 3g). Consistent with previous work^2^, ADRB3-stimulated energy expenditure was lower in mice with loss of *Ckb* from adipocytes (*hM3Dq*^AdipoqCre^:*Ckb*^fl/fl^) compared to control (*hM3Dq*^AdipoqCre^:*Ckb*^+/+^) mice (Extended Data Fig. 9e). Strikingly, combined Gα_s_ and Gα_q_ activation of *hM3Dq*^AdipoqCre^:*Ckb*^fl/+^ mice promoted a sustained rise in energy expenditure above (by about 20%) Gα_s_ signalling alone (Fig. 3j). In contrast, the potentiating effect of Gα_q_ signalling was lost in mice deficient in adipocyte CKB expression (Fig. 3k) and absent in control mice lacking either adipocyte-selective hM3Dq or cre recombinase expression (Extended Data Fig. 9f). Gα_q_ signalling was not sufficient on its own to trigger an elevation of energy expenditure (Extended Data Fig. 9g). Chronic Gα_q_ activation has been reported to inhibit adipocyte differentiation and thus thermogenic output^27^. However, our data suggest that activation of Gα_q_ signalling within mature adipocytes potentiates the acute stimulation of energy expenditure elicited by Gα_s_ signalling in a manner that is genetically dependent on adipocyte *Ckb*.

Next, we explored the adaptive nature of combined Gα_s_ and Gα_q_ signalling (through CL + DCZ administration) on whole-body energy expenditure. Acute activation of Gα_s_ and Gα_q_ signalling in naïve *hM3Dq*^AdipoqCre^:*Ckb*^+/+^ mice elevated energy expenditure above that achieved with individual Gα_s_ signalling (Fig. 3l). Strikingly, daily Gα_s_ and Gα_q_ stimulation promoted a successively higher daily and cumulative energy expenditure compared to single Gα_s_ activation (Fig. 3m,n). In contrast, the adaptive thermogenic output elicited by dual Gα_s_ and Gα_q_ signalling was absent in mice lacking adipocyte *Ckb* (Fig. 3o–q). Thus, *Ckb* is required for the potentiating and adaptive effects of Gα_q_ signalling on energy expenditure in vivo. In mice lacking either adipocyte-selective hM3Dq or cre recombinase expression, no stimulatory effect of Gα_q_ signalling on energy expenditure was observed above sole Gα_s_ activation (Fig. 3r–t). Before any of these drug interventions, there were no differences in energy expenditure (Fig. 3l,m,o,p,r,s), body composition (Extended Data Fig. 9h,i,k,l,n,o) or body weight (Extended Data Fig. 9j,m,p), and physical movement was identical between treatment groups (Extended Data Fig. 9q–s). Hence, combined adipocyte-selective Gα_s_ and Gα_q_ activation causes an adaptive and sustained increase in whole-body energy expenditure that is genetically dependent on the futile creatine cycling effector protein, CKB.

### Thermogenesis by the ADRA1A–Gα_q_–futile creatine cycle axis

**Fig. 4:**
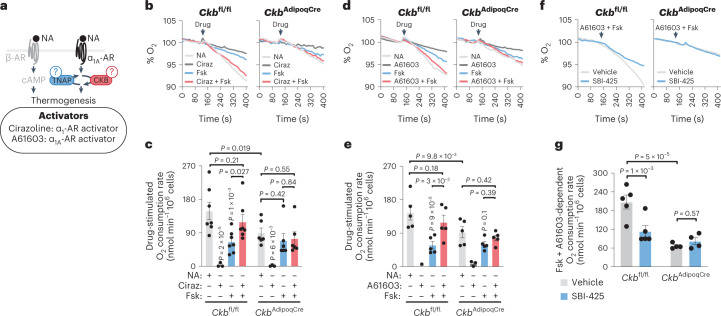
ADRA1A-mediated signalling potentiates thermogenesis through the futile creatine cycle. **a**, Cartoon of approach to study brown adipocyte-intrinsic thermogenesis by individual and combined activation of α_1_-AR or α_1A_-AR with cAMP signalling. **b**,**d**,**f**, Representative oxygen consumption traces of freshly isolated *Ckb*^fl/fl^ and *Ckb*^AdipoqCre^ brown adipocytes. The time of drug addition (arrow) was normalized to 100% for ease of viewing the representative traces. **c**, Oxygen consumption rates of brown adipocytes treated as in **b** (*Ckb*^fl/fl^: *n* = 7, 4, 7 and 7 for NA, Ciraz, Fsk and Ciraz + Fsk, respectively; *Ckb*^AdipoqCre^: *n* = 6, 3, 6 and 6 for NA, Ciraz, Fsk and Ciraz + Fsk, respectively). **e**, Oxygen consumption rates of brown adipocytes treated as in **d** (*n* = 5, 3, 5 and 5 for NA, Ciraz, Fsk and Ciraz + Fsk, respectively for both genotypes). **g**, Oxygen consumption rates of brown adipocytes treated as in **f** (*Ckb*^fl/fl^: *n* = 5; *Ckb*^AdipoqCre^: *n* = 4). Data are presented as the mean ± s.e.m. and *n* indicates the number of biologically independent experiments. **c**,**e**,**g**, Two-way ANOVA (Fisher’s LSD). Source data

**Extended Data Fig. 10:**
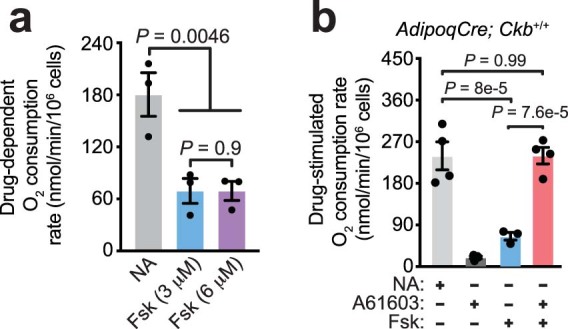
cAMP signaling is not equivalent to NA-stimulated respiration. **a**, Oxygen consumption rates from wild-type (C57BL6/N) brown adipocytes, treated with NA (0.1 μM) or forskolin (Fsk, 3 μM and 6 μM) (*n* = 3 per group). **b**, Oxygen consumption rates of freshly isolated brown adipocytes, treated with NA (0.1 μM), forskolin (Fsk, 3 μM), A61603 (α_1A_AR agonist, 1 μM), or A61603 + Fsk (*n* = 4, *n* = 4, *n* = 3, and *n* = 4 for NA, A61603, Fsk and A61603 + Fsk, respectively). Data are presented as mean ± s.e.m. and *n* numbers are of biologically independent experiments. **a, b**, One-way ANOVA (Fisher’s LSD). Source data

We probed the sufficiency of endogenous adipocyte Gα_q_ signalling (through ADRA1A) to amplify the thermogenic response by cAMP in a brown adipocyte-intrinsic manner (Fig. 4a). The effects of β-AR stimulation in brown adipocytes (lipolysis and thermogenesis) are mediated by cAMP signalling through adenylate cyclase activation. β-AR stimulation can thus, in large, be mimicked by forskolin, a direct activator of adenylate cyclase. However, forskolin could not induce a respiratory response that matched the level achieved by NA in control *Ckb*^fl/fl^ brown adipocytes (Fig. 4b,c), an effect that has previously been reported in brown adipocytes from Syrian hamsters^28^. We confirmed that the maximal rate of forskolin-stimulated respiration had been reached because doubling its concentration did not enhance thermogenesis further (Extended Data Fig. 10a). These data are consistent with the idea that additional signalling mediators other than cAMP regulate thermogenesis downstream of NA engagement to cell surface receptors. Similarly to individual hM3Dq agonism with DCZ, sole α_1_-AR or α_1A_-AR stimulation was not sufficient to trigger oxygen consumption (Fig. 4b–e). In contrast, α_1_-AR or α_1A_-AR stimulation—in the presence of forskolin-mediated cAMP production—potentiated the thermogenic output to a level similar to that achieved by NA in control *Ckb*^fl/fl^ brown adipocytes; however, this response was diminished (by about 80–90%) in *Ckb*^AdipoqCre^ brown adipocytes (Fig. 4b–e). Similarly, inhibition of TNAP activity blocked the capacity for ADRA1A to potentiate thermogenesis in the presence of cAMP production in *Ckb*^fl/fl^ control, but not *Ckb*^AdipoqCre^ brown adipocytes (Fig. 4f,g), indicating that TNAP functions with CKB in tandem to mediate thermogenesis by ADRA1A signalling. Gα_q_ inhibition similarly blocked the potentiating effect of selective ADRA1A stimulation on cAMP-stimulated thermogenesis (Fig. 3e), further demonstrating a functional ADRA1A–Gα_q_ axis in the control of adipocyte thermogenesis. AdipoqCre-expressing *Ckb*^+/+^ brown adipocytes also exhibited a robust capacity for both NA-stimulated and ADRA1A-stimulated potentiation of thermogenesis akin to *Ckb*^fl/fl^ brown adipocytes (Extended Data Fig. 10b), ruling out the possibility that the thermogenic impairments displayed by *Ckb*^AdipoqCre^ brown adipocytes were secondary to cre recombinase toxicity. In aggregate, our data show that in the presence of cAMP production, the ADRA1A–Gα_q_ signalling axis potentiates adipocyte thermogenesis through the futile creatine cycle in a cell-intrinsic manner.

## Discussion

In mouse thermogenic fat, CKB and TNAP promote creatine phosphorylation and phosphocreatine hydrolysis, respectively, to enhance ATP turnover and thermogenesis^2,3^. However, the signal transduction pathways and transcriptional regulators that orchestrate futile creatine cycling gene expression with acute regulation of thermogenesis have remained mysterious. A role for α-AR signalling in rodent and human adipocyte metabolism has been demonstrated previously^28–31^. However, the particular α-AR subtype, the class of G protein that it’s coupled to, and the effector protein(s) that transduce α-AR signalling into a thermogenic output have not been directly demonstrated. Our discovery that the ADRA1A–Gα_q_ signalling axis promotes adipocyte thermogenesis, at least partially, through the futile creatine cycle has several important implications.

First, by uncovering the signalling cascade and effector pathway emanating from ADRA1A stimulation, our study suggests that full adipocyte thermogenic output by NA results from its engagement to both α-ARs and β-ARs, leading to the activation of parallel thermogenic pathways. Because the majority of thermogenic potentiation through ADRA1A was dependent on CKB and TNAP, our data indicate that ADRA1A stimulation potentiates cAMP-induced thermogenesis in a UCP1-independent manner. The identification of distinct adipocyte subtypes^32–36^ and mitochondrial heterogeneity within thermogenic fat cells^37^ suggests that discrete thermogenic pathways could segregate inter-cellularly or intracellularly. *Ckb* and *Alpl* are expressed in *Ucp1*^+^ adipocytes^2,3^, and we demonstrate that genetic or pharmacological inhibition of their protein products decreases thermogenesis in brown adipocytes in the presence of UCP1. These findings suggest that thermogenesis by the futile creatine cycle and by UCP1, at least partly, occur within the same cells. The quantitative contribution of individual thermogenic pathways will be dynamic and will depend on the nature of the extrinsic stimuli and other key factors such as thermal history. However, our findings support the general idea that resolving how individual energy dissipating pathways are coordinated to yield maximal thermogenic output should be a primary goal of the field. This may have important implications for the role of thermogenic fat as a metabolic sink^38,39^ or in tissue cross-talk^40,41^ where both functions are likely to be closely tied to its intrinsic thermogenic properties.

Second, acutely isolated brown adipocytes that we have used in this study are the most physiologically relevant model system for examining cell-autonomous thermogenesis in vitro. Because CKB deficiency or TNAP inhibition individually reduced NA-stimulated respiration to a similar level as ATP synthase blockade in this model system, the futile creatine cycle appears to be a key UCP1-independent thermogenic pathway, at least under the conditions we have used. In our mouse experiments, we combined a chemogenetic approach with CKB loss-of-function models. Our data show that combined Gα_q_ and Gα_s_ signalling promotes whole-body energy expenditure through CKB. CKB is a multi-localized protein, exhibiting mitochondrial and non-mitochondrial targeting in brown adipocytes^2^. Mitochondrial CKB is necessary for thermogenesis by the futile creatine cycle^2^. Non-mitochondrial CKB may support the futile creatine cycle (by regenerating creatine through the reverse creatine kinase reaction) or have a function that is independent of it entirely. For example, it remains to be determined if CKB also supports thermogenesis by maintaining high local ATP/ADP ratios through the phosphocreatine/creatine kinase circuit. Combined activation of Gα_q_ and Gα_s_ signalling in *hM3Dq*^AdipoqCre^ mice genetically altered to lack *Alpl* in adipocytes should help resolve this. The portion of thermogenesis that is similarly reduced between CKB or TNAP loss of function could be attributed to the futile creatine cycle. On the other hand, if CKB simply supports UCP1-mediated uncoupling, by maintaining high ATP/ADP ratios through the phosphocreatine/creatine kinase circuit, CKB should only be required when UCP1 is active. This supposition can be tested with co-deletion of CKB and UCP1 in a mouse model that is not confounded by germline *Ucp1* ablation^42^. Notably, futile creatine cycling and the phosphocreatine/creatine kinase circuit are not mutually exclusive. Future work should address these mechanistic details.

Third, therapeutic targeting of adipocyte thermogenesis will require the effects to be sustained long enough to meaningfully impact nutrient utilization. The in vivo administration of ADRB3 agonists or NA has been the preferred approach to examine adipocyte thermogenesis in preclinical models. We have found that a sustained and adaptive elevation of whole-body energy expenditure could be reached through combined adipocyte-selective Gα_q_ and Gα_s_ signalling. In contrast, with respect to whole-body energy expenditure, ADRB3 signalling alone was not adaptive and NA administration in vivo is not selective to adipocytes and limited to transient thermogenic output. Our data thus indicate that triggering coordinated Gα_q_ and Gα_s_ signalling selectively in adipocytes can aid in the identification of new thermogenic factors, or reveal ones that were missed because thermogenic outputs have been constrained by focusing only on β-AR signalling. Theoretically, orchestrated Gα_q_ and Gα_s_ signalling in adipocytes might be achieved through a diverse combination of non-adrenergic GPCR mechanisms^43,44^ thus potentially circumventing any unwanted side-effects arising from adrenergic stimulation of non-adipocytes. Further research is required to determine if the futile creatine cycle can be activated through a non-adrenergic Gα_q_ manner, which is an important consideration for potential therapeutic targeting of this pathway. Nevertheless, our findings provide a lens for future studies that centre on determining whether safe pharmacological targeting of combined Gα_q_ and Gα_s_ signalling in adipocytes can be achieved. It will be critical to determine whether Gα_q_ potentiates thermogenesis by the Gα_s_ pathway through its regulation over nutrient (glucose) availability in adipocytes, as has recently been demonstrated^45^.

In conclusion, our work has identified a long sought-after effector pathway of α-AR thermogenesis. ADRA1A is an α-AR subtype that is sufficient to potentiate adipocyte thermogenesis through physical and functional coupling to Gα_q_ in mouse adipocytes, and our data imply that the futile creatine cycle is an effector pathway of this process. In addition to defining the features of facultative and adaptive thermogenic activation by combined ADRA1A–Gα_q_ and ADRB3–Gα_s_ signalling, we delineate the transcriptional control of futile creatine cycling genes to be regulated by EBF1/2, ERRα/γ and PGC1α. Thus, while the transcriptional mechanisms regulating *Ucp1* by some of these factors are well characterized^46^, they clearly also play an essential role in the transcriptional regulation of UCP1-independent thermogenesis. We show here that Gα_q_ and Gα_s_ signalling triggers an adaptive and sustained rise in energy expenditure compared to Gα_s_ signalling alone. Our studies centred on the mechanistic aspects of this response, and point to CKB as being a key effector of this process. However, future work should examine whether the level of sustained energy expenditure that we observe with combined Gα_q_ and Gα_s_ signalling is sufficient to improve systemic metabolic parameters in the context of obesity-accelerated glucose intolerance, insulin resistance and tissue inflammation and fibrosis. Whether the capacity for Gα_q_ signalling to promote adipocyte thermogenesis is unique to its potentiating effects on the ADRB3–Gα_s_ axis or is more broadly applicable to other Gα_s_-stimulating factors remains to be seen.

## Methods

### Animals

Mouse experiments were performed according to procedures approved by the Animal Resource Centre at McGill University and complied with guidelines set by the Canadian Council of Animal Care. The photoperiod was fixed at a 12-h light/12-h dark schedule (light 07:00 to 19:00) with lights on at 07:00 h being defined as Zeitgeber time (ZT) 0. Mice had ad libitum access to drinking water and a low-fat diet (3.1 kcal/g energy density) with 24%, 16% and 60% of calories from protein, fat and carbohydrate, respectively (2920X, Envigo). All mice were born and housed in groups (3–5 mice per cage) at 22 °C ± 2 °C at 30–40% humidity with bedding and shredded paper strips in the cage until experimental intervention (6–9 weeks of age). Suitable housing temperature of mice to optimally mimic human physiology is disputed. We followed the suggestion that when provided with bedding and nesting materials, a standard room temperature (22–24 °C) is appropriate^47^. For cold exposure experiments, mice were singly housed in cages with bedding and shredded paper strips and with ad libitum access to drinking water and a low-fat diet. Mouse experiments used age-matched littermates and were conducted at the indicated temperature. Mice were killed by cervical dislocation and tissues were immediately flash frozen in liquid nitrogen and store at −80 °C until further analysis. Wild-type C57BL/6N mice were purchased from Charles River (strain 027). *Ckb*^fl/fl^ mice were previously described^2^. *Ucp1*^CreERT2^ mice^48^ were bred to *Ppargc1a*^fl/fl^ mice to generate experimental groups (*Ppargc1a*^fl/fl^ and *Ppargc1a*^Ucp1CreERT2^). For inducible deletion of *Ppargc1a*, mice were reared at 22 °C ± 2 °C until 7 weeks of age, then injected with tamoxifen (75 mg per kg body weight) for 3 d and allowed to recover for 4 d until intervention. Inducible BAT-specific overexpression of *Gpr3* (I-3BO) and control animals have been previously described^12^. AdipoqCre mice (B6;FVB-Tg(AdipoQ-Cre)1Evdr/J; stock 028020), maintained on a C57BL/6J background, were bred to *Ckb*^fl/fl^ (ref. ^2^), *Ebf1/Ebf12*^fl/fl^ (ref.^16^) and *Esrra*/*Esrrg*^fl/fl^ (refs.^17,49,50^) mice to generate experimental groups *Ckb*^fl/fl^ and *Ckb*^AdipoqCre^, *Ebf1/Ebf2*^fl/fl^ and *Ebf1/Ebf2*^AdipoqCre^ (ref. ^16^) and *Esrra/Esrrg*^fl/fl^ and *Esrra/Esrrg*^AdipoqCre^, respectively. LSL-hM3Dq-DREADD mice (B6N;129-Tg(CAG-CHRM3*,-mCitrine)1Ute/J), stock 026220) were bred to AdipoqCre mice to generate mice that conditionally express a HA-tagged modified muscarinic receptor (HA-hM3Dq) selectively in adipocytes (*hM3Dq*^AdipoqCre^). *hM3Dq*^AdipoqCre^ mice were crossed with *Ckb*^fl/fl^ mice to generate *hM3Dq*^AdipoqCre^:*Ckb*^+/+^, *hM3Dq*^AdipoqCre^:*Ckb*^fl/+^ or *hM3Dq*^AdipoqCre^:*Ckb*^fl/fl^ mice. Sex of mice used for experiments is indicated. Genotyping primer sequences are available in Supplementary Table 1.

### RNA extraction

Total RNA from frozen mouse tissues was extracted using QIAzol (Qiagen) and purified with RNeasy Mini spin columns (Qiagen) according to the manufacturer’s instructions. Total RNA from human tissues, third cohort, was extracted with TRIzol (Gibco BRL, Life Technologies). RNA was quantified using a NanoDrop 8000 Spectrophotometer (Thermo Scientific Pierce). cDNA was synthesized using a Verso cDNA kit (Ab-1453, Thermo Fisher Scientific) with random hexamer primers.

### RT–qPCR

Purified RNA was reverse transcribed using a High-Capacity cDNA Reverse Transcription kit (Applied Biosystems). The resultant cDNA was analysed by RT–qPCR. In brief, 20 ng cDNA and 150 nmol of each primer were mixed with GoTaq qPCR Master Mix (Promega). Reactions were performed in a 384-well format using a CFX384 real-time PCR system (Bio-Rad). Normalized mRNA expression was calculated using the ΔΔCt method, using *Ppib* or *36b4* mRNA as the reference gene. CFX Maestro 2017 was used for data collection. For human samples, PCR reactions were performed in duplicate using LightCycler SYBR Green master mix (Roche Applied Science) in a LightCycler 480 (Roche Applied Science). The following cycling conditions were used: one step at 95 °C for 3 min, then 95 °C for 10 s, 60 °C for 20 s and 72 °C for 10 s, and finally a melting curve analysis was performed. The increase in fluorescence was measured in real time during the extension step. The relative gene expression was estimated using the default ‘Advanced Relative Quantification’ mode of the software version LCS 480 1.5.1.62 (Roche Applied Science) and specificity of the amplification was checked by melting curve analysis. Normalized mRNA expression for human samples was calculated using *PPIA* as the reference gene. Primer sequences used for RT–qPCR can be found in Supplementary Table 2.

### ERRα ChIP–qPCR

For each condition, nuclei were isolated from six individual BAT pads from three male wild-type (C57BL6/N) mice. BAT was dounce homogenized (25 times with pestle A and 15 times with pestle B) in Nuclei Preparation Buffer (10 mM HEPES pH 7.5, 10 mM potassium chloride, 1.5 mM magnesium chloride, 0.1% NP40). BAT homogenate was filtered through 100-μm strainer. Nuclei were fixed with 1% formaldehyde for 12 min at room temperature, quenched by 125 mM of glycine for 10 min and washed twice with 0.1% NP40 in PBS. Chromatin was sonicated in 1 ml of sonication buffer (50 mM Tris-HCl pH 8.1, 10 mM EDTA, 1% SDS) to obtain fragments around 500 base pairs (bp). Then, 20 μg of chromatin DNA was diluted in ChIP Dilution Buffer (16.7 mM Tris-HCl pH 8.1, 1.1% Triton X-100, 167 mM sodium chloride, 1.2 mM EDTA, 0.01% SDS) up to 2 ml. Then, 0.87 μg of anti-ERRα (Abcam, ab76228) was added to the sonicated chromatin and left to rotate overnight at 4 °C. The next day, 50 μl of Dynabeads Protein G (10009D, Thermo Fisher Scientific) was washed twice with PBS + 0.5% TWEEN and 0.5% BSA and added to the chromatin for 1 h under rotation at 4 °C. Next, Dynabeads were washed twice with 1 ml of cold Low Salt RIPA Buffer (0.1% SDS, 1% Triton X-100, 1 mM EDTA, 20 mM Tris-HCl pH 8.1, 140 mM sodium chloride, 0.1% sodium deoxycholate), twice with 1 ml of cold High Salt RIPA Buffer (0.1% SDS, 1% Triton X-100, 1 mM EDTA, 20 mM Tris-HCl pH 8.1, 500 mM sodium chloride, 0.1% sodium deoxycholate), twice with 1 ml of cold lithium chloride wash buffer (250 mM lithium chloride, 0.5% NP40, 0.5% sodium deoxycholate, 1 mM EDTA,10 mM Tris-HCl pH 8.1) and twice with room-temperature TE buffer (10 mM Tris-HCl pH 8.0, 1 mM EDTA pH 8.0). DNA was eluted overnight at 65 °C with 100 μl of ChIP Elution Buffer (10 mM Tris-HCl pH 8.0, 5 mM EDTA, 300 mM sodium chloride, 0.1% SDS, 5 mM dithiothreitol) and 16 μl Reverse Cross-Linking mix (250 mM Tris-HCl pH 6.5, 62.5 mM EDTA pH 8.0, 1.25 M sodium chloride, 5 mg ml^−1^ Proteinase K, 62.5 μg ml^−1^ RNase A). Finally, immunoprecipitated chromatin DNA was purified using a QIAquick PCR purification kit and eluted in 31 μl of Elution buffer (10 mM Tris-HCl pH 8.0, 0.1 mM EDTA pH 8.0). Relative ChIP fold enrichments were controlled by inputs and normalized to the average of two non-specific control regions using a LightCycler 480 (Roche) using SYBR Green I Master Mix (4887352001, Roche) as previously published^51^. Results represent the average of three replicates. Gene-specific and non-specific control primers used for ChIP–qPCR analysis can be found in Supplementary Table 3.

### Administration of chemicals by osmotic pump

Osmotic pumps (Alzet) were loaded with CL 316,243 in 100 μl total volume. Mice were anaesthetized with isoflurane. A small skin incision was made directly above the iBAT depot. Filled osmotic pumps were placed above the iBAT and the skin was then sewed. Tissues were collected for gene expression analyses following intervention. The release rate from the osmotic pumps is 0.5 μl per hour, so, as an example, loading 0.5 mg of CL 316,243 in 100 μl for a 24 g mouse will administer approximately 0.1 mg per kg body weight CL 316,243 every hour, totalling 5 mg per kg body weight over a 48-h treatment.

### Administration of chemicals by intraperitoneal injection

Mice were reared at 22 °C, housed at 30 °C for 5 d before drug injections (unless otherwise noted). PBZ was dissolved in saline and was injected (5 mg per kg body weight) once over 24 h. PZS was dissolved in 4% DMSO, 30% PEG and 66% saline and was injected (5 mg per kg body weight) three times over 24 h. Propranolol hydrochloride (Prop) was dissolved in saline and injected (5 or 10 mg per kg body weight) three times over 24 h. All inhibitors were initially injected i.p. at ZT 3 at 1 h before cold exposure. For CL 316,243 treatment, mice were injected (five injections over 48 h) i.p. with CL 316,243 (1 mg per kg body weight) or an equivalent volume of saline as control.

### Cold exposure

Mice were reared at 22 °C, housed at 30 °C for 5 d before cold exposure (unless otherwise noted). The precise temperature and time spent in the cold is noted.

### Animal treatments for RNA-seq and ATAC–seq

Wild-type male mice (C57BL6/N, 6–8 weeks of age) were reared at 22 °C, housed at 30 °C for 5 d and then subjected to 30 °C or 6 °C at ZT4 (*n* = 3 per group). One hour before onset of 6 °C exposure (ZT3), mice were injected i.p. with PBZ (5 mg per kg body weight) or saline (Sal). BAT was harvested 24 h after onset of 6 °C exposure for downstream analysis by RNA-seq and ATAC–seq.

### RNA-seq analysis

Total RNA was quantified using a NanoDrop Spectrophotometer ND-1000 (NanoDrop Technologies) and its integrity was assessed on a 2100 Bioanalyzer (Agilent Technologies). Libraries were generated from 250 ng of total RNA as follows: mRNA enrichment was performed using the NEBNext Poly(A) Magnetic Isolation Module (New England BioLabs). cDNA synthesis was achieved with the NEBNext RNA First Strand Synthesis and NEBNext Ultra Directional RNA Second Strand Synthesis Modules (New England BioLabs). The remaining steps of library preparation were done using the NEBNext Ultra II DNA Library Prep Kit for Illumina (New England BioLabs). Adaptors and PCR primers were purchased from New England BioLabs. Libraries were quantified using the Kapa Illumina GA with Revised Primers-SYBR Fast Universal kit (Kapa Biosystems). Average size fragment was determined using a LabChip GX (PerkinElmer) instrument. The libraries were normalized and pooled and then denatured in 0.05 N sodium hydroxide and neutralized using HT1 buffer. The pool was loaded at 225 pM on an Illumina NovaSeq S4 lane using Xp protocol according to the manufacturer’s recommendations. The run was performed for 2 × 100 cycles (paired-end mode). A phiX library was used as a control and mixed with libraries at the 1% level. Base calling was performed with RTA (v3.4.4). The programme bcl2fastq2 (v2.20) was then used to demultiplex samples and generate fastq reads. Adaptor sequences and low-quality score bases (Phred score < 30) were first trimmed using Trimmomatic (v.0.36)^52^. The resulting reads were aligned to the GRCm38 mouse reference genome assembly, using STAR (v.2.0.2)^53^. Read counts were obtained using HTSeq (v.0.6.0)^54^ with the parameters --m intersection--nonempty --stranded = reverse. For all downstream analyses, we excluded genes with low expression levels that had an average read count lower than 10 across all samples, resulting in 17,952 genes in total. Raw counts were normalized using edgeR’s TMM algorithm (v3.26.8)^55^ and were then transformed to log_2_CPM using the voom function implemented in the limma R package (v4.2)^56^. To assess differences in gene expression levels between the different conditions, we fitted a linear model using limma’s lmfit function. Nominal *P* values were corrected for multiple testing using the Benjamini–Hochberg method. To specifically identify temperature-sensitive genes that further respond differently to PBZ treatment, we first obtained DEGs in 6 °C versus 6 °C + PBZ (FDR < 0.1) and then filtered for those that change expression in comparison to 30 °C (that is, in either 6 °C versus 30 °C or 6 °C + PBZ versus 30 °C; FDR < 0.01 and |log2FC| > 1). Unsupervised hierarchical clustering of the 764 DEGs showed four distinct patterns of changes in expression (R hclust function). The complete list of DEGs and their cluster annotation is presented in Supplementary Data 1. Enrichment analyses of GO Biological Processes were performed using Enrichr^57^. The enrichment results for cluster 4 are reported in Extended Data Fig. 2d.

### Generation of ATAC–seq libraries from brown adipose tissue

For each condition, nuclei were isolated from four individual frozen BAT pads from two male wild-type (C57BL6/N) mice. Animals were housed at 30 °C for 5 d then exposed to cold (6 °C) or kept at 30 °C for 24 h. Mice were injected with vehicle (0.9% saline) or PBZ (5 mg per kg body weight) 1 h before cold exposure. Our protocol and the buffers used were adapted from work by ref. ^58^ with some modifications. Briefly, for nuclei preparation, BAT pads were homogenized (with pestle A) in a pre-chilled 2 ml Dounce homogenizer containing 2 ml cold 1× Homogenization Buffer (60 mM Tris pH 7.8, 30 mM CaCl_2_, 18 mM magnesium acetate, 320 mM sucrose, 0.1 mM EDTA, 0.1% NP40, 0.1 mM PMSF, 1 mM β-mercaptoethanol). The resulting solution was pre-cleared using a 100-μm filter and grounded 20 times (with pestle B). To generate the iodixanol gradient, one volume (800 μl) of 50% iodixanol solution was added to 800 μl of grounded BAT solution to give a final concentration of 25% iodixanol in a 5-ml low-bind microcentrifuge tube. Then, 1.2 ml of 29% iodixanol solution was added under the 25% mixture, and another 1.2 ml of 35% iodixanol solution wad added under the 29% mixture. The gradient was centrifuged at 3,000*g* for 20 min at 4 °C with the brake off. The nuclei band was collected into a new tube and nuclei were counted using trypan blue staining and a Countess II FL automated cell counter. Around 50,000 nuclei were transferred into a tube containing 1 ml of ATAC-RSB + 0.1% Tween-20 and centrifuged at 500*g* for 10 min at 4 °C. For optimized transposition, Omni-ATAC ATAC–seq reaction mix (25 μl 2× TD buffer, 100 nM transposase, 16.5 μl PBS, 0.01% digitonin, 0.1% Tween-20)^58^ was added to the nuclei pellet. Nuclei were resuspended by pipetting up and down six times. The resulting solution was incubated at 37 °C for 30 min in a thermomixer (1,000 r.p.m.). For the pre-amplification of transposed fragments, the solution was cleaned with a Zymo DNA Clean and Concentrator-5 kit (D4014) according to the manufacturer’s instructions. DNA was eluted in 21 μl elution buffer and amplified for five cycles using NEBNext 2× MasterMix (NEB, M0541L) as previously described^58^ using published primers^59^. Then, 10% of the pre-amplified mixture was used to run qPCR to determine the number of additional cycles needed as previously described^58^. Next, the amplification profiles were manually assessed and the required number of additional cycles were determined as previously described^60^. The final PCR reaction was purified using a Qiagen MinElute PCR Purification Kit and eluted in 20 μl elution buffer. A subsample of each library was diluted to a ratio of 1:1,000 to fall within range of the standards to perform concentration quantification using the KAPA Library Quantification Kit (KK4854) according to the manufacturer’s instructions. Paired-end, 150-bp sequencing was performed on a HiSeq instrument at the Michael Smith Genome Sciences Centre (BC Cancer Research Institute).

### ATAC–seq analysis

ATAC–seq reads were first trimmed for adaptor sequences and low-quality score bases using Trimmomatic^52^. The resulting reads were mapped to the mouse reference genome (mm10) using BWA-MEM (v.0.7.12)^61^ in paired-end mode using default parameters. Only reads that had a unique alignment (mapping quality > 20) were retained and PCR duplicates were marked using Picard tools (v.2.0.1; https://broadinstitute.github.io/picard/). Peaks were called and annotated using MACS2 (v.2.1.1.2060309)^62^. Peak annotation and transcription factor motif enrichment analysis were performed using the annotatePeaks and findMotifsGenome commands, respectively, from HOMER software suite^63^. Peaks were associated to a gene if located within 20 kb of the transcription start site. To assess differences in chromatin accessibility, a ‘reference peak set’ was generated by merging ATAC–seq peaks across samples, using bedtools merge with the parameters: --sorted --d --125 (https://bedtools.readthedocs.io/). Read counts were obtained within these genomic regions using HOMER (v.4.9.1). Raw counts were normalized using edgeR’s TMM algorithm^55^ and were then transformed to log_2_CPM using the voom function implemented in the limma R package (v.3.40.6)^56^. To test for differential occupancy, we fitted a linear model that takes into account the batch effects in the experiment. Nominal *P* values were corrected for multiple testing using the Benjamini–Hochberg method. Read density metagene plots and heat maps were obtained using ngs.plot^64^ with the following parameters --G mm10 --BOX 0 --SE 0 --VLN 0 --LWD 2 --WD 9 --L 1500. Genome browser tracks were created with the HOMER makeUCSCfile command and bedGraphToBigWig utility from the University of California, Santa Cruz (UCSC). Tracks were normalized so that each value represents the read count per bp per 10 million reads. UCSC Genome Browser (https://genome.ucsc.edu/) was implemented for track visualization.

### Western blotting

Samples were prepared in lysis buffer (50 mM Tris, pH 7.4, 500 mM sodium chloride, 1% NP40, 20% glycerol, 5 mM EDTA and 1 mM PMSF), supplemented with a cocktail of Roche protease inhibitors. The homogenates were centrifuged at 16,000*g* for 10 min at 4 °C, and the supernatants were used for subsequent analyses. Protein concentration was determined using the bicinchoninic acid assay (Pierce). The quantity of protein lysate to use for each antibody was determined empirically. Protein lysates were denatured in Laemmli buffer (60 mM Tris, pH 6.8, 2% SDS, 10% glycerol, 0.05% bromophenol blue, 0.7 M β-mercaptoethanol), resolved by 10% Tris/Glycine SDS–PAGE and transferred to a polyvinylidene difluoride membrane. Primary antibodies were diluted in TBS containing 0.05% Tween, 5% BSA and 0.02% sodium azide. Membranes were incubated overnight with primary antibodies at 4 °C. Primary and secondary antibody dilutions can be found in the Reporting Summary. Results were visualized with enhanced chemiluminescence western blotting substrates (Bio-Rad).

### Indirect calorimetry

Mice had ad libitum access to drinking water and a low-fat diet (2920X, Envigo). All mice used for indirect calorimetry experiments were born and housed in groups (3–5 mice per cage) at 22 °C with bedding and shredded paper strips in the cage until experimental intervention. Mice (6–8 weeks of age) were placed, single housed, in metabolic cages (Sable Systems International, Promethion high-definition behavioural phenotyping system) housed in thermal cabinets set to 30 °C with a 12-h light/12-h dark schedule (light 07:00 to 19:00). Mice had ad libitum access to food and water and were allowed to acclimatize to 30 °C for 5 d. The following morning between ZT2 and ZT3, mice were injected with vehicle (saline) and placed back in the metabolic cages to monitor the stress response to i.p. injection. The next morning (at ZT2–3), the same volume of the β_3_-adrenoreceptor agonist CL 316,243 (0.5 mg per kg body weight) or CL 316,243 + DCZ (0.5 mg per kg body weight each) was administered i.p. and mice were placed back in the metabolic cages. For chronic daily drug injections mice were injected daily at the same time (at ZT2–3) with CL 316,243 (0.5 mg per kg body weight) or CL 316,243 + DCZ (0.5 mg per kg body weight each) for 7 d. For NA experiments, NA (Sigma, A9512) was prepared fresh in saline and administered i.p. at 1 mg per kg body weight at 30 °C. Responses to drugs were followed every 3 min. Mass-dependent variables (energy expenditure) was not normalized to body weight. Energy expenditure (kcal), physical movement (measured by infrared beam breaks), and food intake were recorded every 3 min using Sable Systems data acquisition software (IM-3 v.20.0.3). Data were analysed using Sable Systems International Macro Interpreter software (v.2.41) using One-Click Macro (v.2.37).

### Isolation of brown adipocytes

Interscapular BAT was minced and digested in a Krebs-Ringer bicarbonate modified buffer (135 mM sodium chloride, 5 mM potassium chloride, 1 mM calcium chloride, 1 mM Magnesium chloride, 0.4 mM dipotassium phosphate, 25 mM sodium bicarbonate, 20 mM HEPES, 10 mM glucose, 4% fatty-acid-free BSA), supplemented with 2 mg ml^−1^ collagenase B (Worthington) and 1 mg ml^−1^ soybean trypsin inhibitor (Worthington). Minced BAT from ten mice was digested in 20 ml Krebs-Ringer digestion buffer with continuous shaking at 37 °C for 45 min. The tissue suspension was filtered through a 100-μm cell strainer. Brown adipocytes were allowed to float for 5 min at room temperature before and after spinning at 200*g* for 5 min. Half of the infranatant was removed (~10 ml) with a 20-ml syringe/18-gauge needle, followed by the removal of the stromal vascular fraction. Adipocytes were washed with 10 ml DMEM/F12 supplemented with 10% FBS and were allowed to float for 20 min at room temperature before spinning at 200*g* for 5 min. Adipocytes were washed three times. After the final wash, the mature adipocytes present under the fat layer were transferred to a new tube. Cell number was determined using a Bright-Line Hemacytometer (Hausser Scientific).

### Respirometry of purified adipocytes using an oxygen electrode

A Clark-type electrode (Rank Brothers) was used to measure the oxygen consumption of adipocytes. DMEM/F12 supplied with 10% FBS was added to the chamber and left to equilibrate with atmospheric oxygen. Approximately 10,000 cells were then added to the chamber (0.7 ml final volume), covered with a lid and continuously stirred. The initial rate of cellular respiration before the addition of a thermogenic activator was termed ‘basal respiration’. Thermogenic drugs were added to the continuously stirring cells via a Hamilton syringe (0.1 µM NA, 3 µM forskolin, 1 µM cirazoline, 1 µM A61603). Inhibitors were added to the respiration buffer before the addition of cells at the following final concentrations: 1 µM PBZ, 1 µM PZS, 10 µM RS-17053, 10 µM YM-254890 and 10 µM SBI-425. Oligomycin (5 µM) was injected acutely following NA. To measure the acute effect of different drugs on respiration, the linear portion of the oxygen consumption rates was measured. The excess of oxygen consumed after the addition of the drugs was subtracted from the basal respiration rate to quantify the drug-dependent oxygen consumption. To plot the representative traces, the time of drug addition was normalized to 100% to simplify comparison of the different groups. For some traces, the addition of the drugs caused an artificial increase in oxygen levels inside the chambers. This increase was subtracted in the representative traces to avoid confusion with the actual effect of the drugs and simplify their visualization. Importantly, no normalization was applied to the calculation of the oxygen consumption rates shown in the bar graphs. Multiple electrodes were used simultaneously to measure respiratory effects of distinct treatment groups in parallel, and different treatments were switched between electrodes to avoid any potential systematic bias (starting oxygen concentration between treatment groups/genotypes) coming from a single electrode. Rank Brothers Dual Digital model 20: Picolog 6 data logging software was used for data collection.

### Unilateral denervation of interscapular brown adipose tissue

Unilateral denervation was carried out as previously described^12^. Briefly, 22 °C-housed mice were anaesthetized by inhalation of isoflurane (2.5% for induction, 1.5% for maintenance) and the incision site was shaved and disinfected first by using 0.5% chlorohexidine in 85% ethanol and then 70% ethanol. Before surgery, mice received local anaesthesia (lidocaine, 1.4 mg per kg body weight) and general analgesia (Rimadyl, 10 mg per kg body weight). The iBAT was prepared by a midline incision of the skin in the interscapular region and the detachment of the iBAT from the underlying muscle layer. The five nerve fibres innervating the right iBAT lobe were identified and cut (denervated), and the nerve fibres innervating the left iBAT lobe were identified and touched with forceps (sham). Following the procedure, the fat pads were rinsed with sterile isotonic saline and the incision was closed with suture. The mice were individually housed in clean cages at 22 °C with access to a 37 °C heating pad during the first 24 h after the operation. Animals were monitored daily.

### Glycerol release

Freshly isolated brown adipocytes (15,000 cells in 0.3 ml) in DMEM/F12 (Thermo Fisher Scientific) supplemented with 4% fatty-acid-free BSA and were incubated with 0.1 μM NA, 1 μM PBZ or 10 µM SBI-425 for 1 h at 37 °C. Following incubation, released glycerol was separated from the adipocytes by spinning through a centrifugal filter (Millipore sigma, UFC30LG25) at 8,000*g* for 30 s at room temperature. The glycerol content in the medium was determined using free glycerol reagent (Sigma, F6428) and glycerol standard solution (Sigma, G7793) according to the manufacturer’s instructions.

### Plasmids

*Adra1a-Nluc* was generated by amplifying the full-length sequence of *Nluc* (including linker) from *Gpr120-Nluc* (forward: 5′-GAG GAA GTC TCG GAA TTC GCC GCC ATG GTC TTC-3′; reverse: 5′-ACC CTT TTA CGC CAG AAT GCG TTC GCA CAG C-3′) and fusing it in frame to untagged *Adra1a* by amplifying the vector encoding *Adra1a* without its stop codon through the use of PCR (forward: 5′-CAT TCT GGC GTA AAA GGG TGG GCG CGC CGA CC-3′ and reverse: 5′-GAA TTC CGA GAC TTC CTC CCC GTT TTC ACC GAG-3′) and Gibson assembly. *Adra1a-Nluc* was PCR amplified (forward: 5′-GAT ACC GGA TCC GCG ACG ATG GTG CTT CTT TCT GAA-3′ and reverse: 5′-TGC TTA CTC GAG TTA CGC CAG AAT GCG TTC-3′) and subcloned into pcDNA3 via BamH1 and Xho1 restriction sites.

### Bioluminescence resonance energy transfer-based miniG subtype recruitment

Immortalized mouse brown preadipocytes were grown with DMEM with 10% FBS and penicillin–streptomycin. Following confluency, cells were differentiated with DMEM containing a differentiation cocktail of 20 nM insulin, 1 μM dexamethasone, 0.5 μM rosiglitazone, 1 nM T3 and 500 μM methyl isobutyl xanthine. After 2 d of differentiation, medium was replaced with DMEM with 10% FBS containing 1 nM T3 and 20 nM insulin. The next day, the differentiated adipocytes were transfected using TransIT-X2 (Mirus), per the manufacturer’s protocol. Briefly, plasmid DNA encoding *Adra1a-NanoLuc*, as well as venus-tagged miniG protein subtypes (miniG_i_, miniG_s_, miniG_q_, miniG_12_ and miniG_o_; provided by N. Lambert, Augusta University) were added to a sterile tube containing OptiMEM and TransIT-X2. The TransIT-X2:DNA complexes were plated into selected wells of a 96-well white polystyrene Nunc microplate (Sigma) and left to incubate at room temperature for 15–30 min. Differentiated adipocytes were trypsinized and resuspended in DMEM with 10% FBS and applied to selected wells at a density of 60,000 cells per well and incubated overnight.

For BRET experiments, 24 h after transfection, differentiated adipocyte medium was replaced with HBSS supplemented with 10 mM HEPES, pH 7.5, and 10 μM furimazine (NanoGlo, Promega). BRET measurements were performed at 37 °C using a PHERAstar Microplate Reader (BMG Labtech) with a dual-luminescence readout BRET1 plus filter (donor wavelength, 460–490 nm band pass; acceptor wavelength, 520–550 nm long pass). Following four baseline measurements, the cells were treated with vehicle or agonists (NA, cirazoline or A61603, all used at 1 μM) in triplicate for each condition, with the BRET signal measured every 2 min for 1 h. The BRET ratio (acceptor 520–550 nm emission over donor 460–490 nm emission) was calculated for each well over time. The resulting ligand-induced BRET ratio was calculated by subtracting the baseline vehicle read from the agonist-stimulated read for each condition.

### Human studies

In this report, we used human adipose tissue biopsy samples collected from three independent cohorts.

#### First cohort—Joslin Diabetes Center adipose tissue cohort

Details on the procedures of human adipose tissue biopsy sample collection have been described previously^65,66^. Briefly, ten paired human neck fat samples were obtained from superficial SAT depots and deep BAT tissue located proximal to the carotid sheath (*n* = 10 for each tissue). These volunteers (aged 49 ± 12.6 years) were typically being treated for cervical spine stenosis, causing radiculopathy or myelopathy. The volunteers did not undergo metabolic or physiological testing in conjunction with their spinal treatment. Tissue processing, RNA isolation and analysis of gene expression has been previously described^65^. Briefly, analysis of gene expression using GeneChIP PrimeView (Affymetrix) was performed on matched biopsy samples as previously described^66^. RNA was isolated from clonal cell lines using Direct-zol RNA MiniPrep kit (Zymo Research) according to the manufacturer’s instructions. The quality of total RNA was evaluated by an A260/A280 ratio, which was within the value of 1.9 to 2.0 defined as high-quality total RNA. Biotin-labelled cRNA was synthesized, purified and fragmented using GeneChip 3′ IVT Express Kit (Affymetrix). Integrity and fragmented cRNA was assessed by running aliquots on the Agilent 2100 Bioanalyzer before proceeding further. High-quality cRNA meets the following criteria: the A260/A280 ratio should fall within the value of 1.9 to 2.0; the 28S/18S RNA bands (from the gel) should be crisp and the intensity of the 28S band should be roughly twice the intensity of the 18S band. Array hybridization and scanning were performed by the Advanced Genomics and Genetics Core of Joslin Diabetes Center according to established methods. Microarray data were normalized using robust multi-array average, which placed it on a log_2_ scale. All volunteers provided informed consent before taking part in the study. This study followed the institutional guidelines of and was approved by the Human Studies Institutional Review Boards of Beth Israel Deaconess Medical Center and Joslin Diabetes Center.

#### Second cohort—University of Texas Medical Branch Washington University adipose tissue cohort

Twenty-three men and women with overweight or obesity (age, 41 ± 12 years; body mass index, 31.0 ± 3 kg/m^2^) were enrolled in two clinical trials (NCT02786251 and NCT01791114) performed to determine the role of BAT in metabolic regulation in people. All participants completed a comprehensive screening evaluation that included a medical history and physical examination, standard blood tests and an oral glucose tolerance test. Potential participants were excluded if they had diabetes or other serious diseases, smoked cigarettes, consumed excessive alcohol, were pregnant or lactating, or had metal implants that interfered with the imaging procedures. The studies were approved by the Institutional Review Board of the University of Texas Medical Branch in Galveston and the Washington University School of Medicine in St. Louis. Written informed consent was obtained from all volunteers before their participation. Each participant completed a cold exposure study visit to assess BAT volume and activity and to obtain supraclavicular adipose tissue biopsy samples. During this visit, a standard cooling protocol was performed to maximize non-shivering thermogenesis^67,68^. After 6 h of mild exposure to cold (~20 °C), an ^18^F-fluoro-deoxy-glucose positron emission tomography-computed tomography scan was performed to determine BAT characteristics (volume and activity)^67^. Adipose tissue samples from the supraclavicular area—where BAT is primary localized in humans—obtained using a positron emission tomography-computed tomography-guided percutaneous needle biopsy technique^69^.

For adipose tissue processing and RNA-seq analysis, approximately 100 mg of adipose tissue was used for extraction of RNA using the RNeasy Lipid Tissue Mini Kit (Qiagen) including an on-column DNase digestion step. RNA-seq libraries were generated using the Illumina TruSeq Stranded Total RNA Library Prep Gold with TruSeq Unique Dual Indexes (Illumina). Samples were processed following the manufacturer’s instructions, except for modifying the RNA shear time to 5 min. Resulting libraries were multiplexed and sequenced with 75-bp single reads (SR75) to a depth of approximately 25 million reads per sample on an Illumina HiSeq 4000. Samples were demultiplexed using bcl2fastq v2.20 Conversion Software (Illumina).

#### Third cohort—Danish adult neck adipose tissue cohort

Adipose tissue biopsy samples from the superficial (subcutaneous and subplatysmal) neck fat and deep (carotid sheath, longus colli and prevertebral) neck fat were collected during surgery (age, 58.1 ± 12.9 years; weight, 77.8 ± 17.4 kg; height, 171.4 ± 8.57 cm), as previously described^70^. None of the participants had diabetes nor were they administered β-adrenergic antagonists. All biopsy samples were collected during winter and early spring and were instantly frozen in liquid nitrogen. Only paired biopsy samples from SAT and BAT of the same participants were used for associations (*n* = 73). All study participants gave informed written consent. The study was approved by the Central Denmark Region ethics committee and was performed in accordance with the Declaration of Helsinki. *CKB*, *ALPL*, *ADRA1A* and *UCP1* mRNA expression was analysed using RT–qPCR as described above.

### Statistical analyses

Statistical analyses were performed with GraphPad Prism 9. Data analysis was performed using Microsoft Office Excel 2021 (v.16.56). Data were expressed as the mean ± s.e.m. Unpaired two-tailed Student’s *t*-test for pairwise comparison, one-way ANOVA and two-way ANOVA for multiple comparisons involving two independent variables and Pearson correlation for linear regression, were used to calculate *P* values to determine statistical differences. ANCOVA was used for in vivo metabolic analyses using body weight obtained at the end of the treatment as the covariate. Significance was considered as *P* < 0.05. Actual *P* values are reported. Mice were randomly assigned to treatment groups for in vivo studies. *n* values represent independent biological replicates for cell experiments or individual mice for in vivo experiments.

### Reporting summary

Further information on research design is available in the Nature Research Reporting Summary linked to this article.

## Supplementary information

Supplementary Information Supplementary Tables 1–3

Reporting Summary

Supplementary Data 1 List of genes in the individual clusters from the heat map in Fig. 1d.

## Data Availability

RNA-seq and ATAC–seq data are available in NCBI’s Gene Expression Omnibus under accession number GSE207342. Source data are provided with this paper.

## Source data

Source Data Fig. 1 Statistical source data.
Source Data Fig. 1 Unprocessed western blots.
Source Data Fig. 2 Statistical source data.
Source Data Fig. 3 Statistical source data.
Source Data Fig. 4 Statistical source data.
Source Data Extended Data Fig. 1 Statistical source data.
Source Data Extended Data Fig. 2 Statistical source data.
Source Data Extended Data Fig. 2 Unprocessed western blots.
Source Data Extended Data Fig. 3 Statistical source data.
Source Data Extended Data Fig. 3 Unprocessed western blots.
Source Data Extended Data Fig. 4 Statistical source data.
Source Data Extended Data Fig. 4 Unprocessed western blots.
Source Data Extended Data Fig. 5 Statistical source data.
Source Data Extended Data Fig. 6 Statistical source data.
Source Data Extended Data Fig. 6 Unprocessed western blots.
Source Data Extended Data Fig. 7 Statistical source data.
Source Data Extended Data Fig. 8 Statistical source data.
Source Data Extended Data Fig. 9 Statistical source data.
Source Data Extended Data Fig. 9 Unprocessed western blots.
Source Data Extended Data Fig. 10 Statistical source data.
