# Supplementary material for: ADRA1A–Gαq signalling potentiates adipocyte thermogenesis through CKB and TNAP
Source: Nat Metab. 2022 Nov 7;4(11):1459–73. doi: 10.1038/s42255-022-00667-w (PMC9684074; doi:10.1038/s42255-022-00667-w)
Supplement: Supplementary file 1 — Supplementary Tables 1–3 [file 42255_2022_667_MOESM1_ESM.pdf]

nature metabolism

Letter

https://doi.org/10.1038/s42255-022-00667-w

ADRA1A–Gα_q signalling potentiates adipocyte thermogenesis through CKB and TNAP

In the format provided by the authors and unedited

Supplementary Table 1

Genotyping oligonucleotides	
	Sequences
**Ckb (WT and conditional alleles):**	
Ckb forward	AGG TGG TGG CTA GAG TGA GC
Ckb reverse	CAA GGA TCC CAC TGC TCT TC
***Adrb1*:**	
Adrb1^{+} forward	TCG CTA CCA GAG TTT GCT GA
Adrb1^{+} reverse	GGC ACG TAG AAG GAG ACG AC
Adrb1^{-} forward	TCG CCT TCT TGA CGA GTT CT
Adrb1^{-} reverse	TGG CTC TCT ACA CCT TGG AC
**Ebf1/Ebf2 (WT and conditional alleles):**	
Ebf1 forward	CCC CCA CTT CTT GAG GCC TGT GTT GC
Ebf1 reverse	CCT GGG AGT GGA GGG AGG AAG GAA AAT
Ebf2 forward	GAA CTT GGG GCC AGC CTT TT
Ebf2 reverse	TGT CCT TGA GCT CCC AGG CT
**Essra/Essrg (WT and conditional alleles):**	
Essra forward	ATG AGC CAG GAT GCA GGT GCC TGC A
Essra reverse	GGC TGA GGC AAA GGG ATG GAG GGG T
Essrg forward	GTT TTA AAG GCC CTT GGT GAT CTC GC
Essrg reverse	CTG CAA CCC TTG GAC TGC CAG AAC
**Ppargc1a (WT and conditional alleles):**	
Ppargc1a forward	TCC AGT AGG CAG AGA TTT ATG AC
Ppargc1a reverse	TGT CTG GTT TGA CAA TCT GCT AGG TC
**CAG-LSL-Gq-DREAD:**	
GqDREADD forward	CGC CAC CAT GTA CCC ATA C
GqDREADD reverse	GTG GTA CCG TCT GGA GAG GA
**AdipoqCre:**	
AdipoqCre forward	ACG GAC AGA AGC ATT TTC CA
AdipoqCre reverse	GGA TGT GCC ATG TGA GTC TG
**Ucp1CreERT2:**	
Ucp1CreERT2 forward	GAA CCT GAT GGA CAT GTT CAG G
Ucp1CreERT2 reverse	AGT GCG TTC GAA CGC TAG AGC CTG T

Supplementary Table 2

RT-qPCR oligonucleotides (mouse)	
	Sequences
Ckb forward	GCC TCA CTC AGA TCG AAA CTC
Ckb reverse	GGC ATG TGA GGA TGT AGC CC
Alpl forward	CCA ACT CTT TTG TGC CAG AGA
Alpl reverse	GGC TAC ATT GGT GTT GAG CTT TT
Ppargc1a forward	AGC CGT GAC CAC TGA CAA CGA G
Ppargc1a reverse	GCT GCA TGG TTC TGA GTG CTA AG
Esrra forward	CCA GAG GTG GAC CCT TTG CCT TTC
Esrra reverse	CAC CAG CAG ATG CGA CAC CAG AG
Esrrg forward	CTC CAG CAC CAT CGT AGA GGA TC
Esrrg reverse	GAT CTC ACA TTC ATT CGT GGC TG
Ucp1 forward	AAG CTG TGC GAT GTC CAT GT
Ucp1 reverse	AAG CCA CAA ACC CTT TGA AAA
Ebf1 forward	CCA ACA GCG AAA AGA CCA ATA A
Ebf1 reverse	TGT TCT GTC CGT ATC CCA TTG
Ebf2 forward	CCA GCT CTG AAA GAC AAG TCG
Ebf2 reverse	GAG GTT GCT TTT CAA AAT GGG C
Ppib forward	GGA GAT GGC ACA GGA GGA A
Ppib reverse	GCC CGT AGT GCT TCA GCT T
36b4 forward	TCA TCC AGC AGG TGT TTG ACA
36b4 reverse	GGC ACC GAG GCA ACA GTT
**RT-qPCR oligonucleotides (human)**	
	Sequences
CKB forward	ACT TCA GAA GCG AGG CAC AG
CKB reverse	GAT GAG CAG CTT CAC TCC GT
ADRA1A forward	AAT GCT TCC GAC AGC TCC AA
ADRA1A reverse	TAG TGC GTG ACT GAG TGC AG
ADRB1 forward	GTG GCC CTG CGC GAG CAG AAG
ADRB1 reverse	GCG TAG CCC AGC CAG TTG AAG A
ADRB3 forward	CTC CCC TGG TTC CAT TCC TT
ADRB3 reverse	TGG TCT TTT CTA CCC TGC TGC
PPIA forward	TCC TGG CAT CTT GTC CAT
PPIA reverse	TGC TGG TCT TGC CAT TCC T

Supplementary Table 3

ChIP-qPCR oligonucleotides (mouse)	
	Sequences
ERRα -550 (positive control)	
ERRα -550 forward	GTG GCC CCG CCT TTC CCC GTG ACC TTC ATT
ERRα -550 reverse	ACC CCT GAG GAC CCT CAA GTG GAG AAG CAG
ERRα -3722 (negative control)	
ERRα -3722 forward	TTG GCA TTG ATA TTG GGG GTG GGA GCA ACT
ERRα -3722 reverse	GAC TTC TTA CTT TGA CGC TTT CCT CCA TCG
Prox1 -55772 (negative control)	
Prox1 -55772 forward	CCA AGC ACA AAT ATC TAA TCA CCC TTT C
Prox1 -55772 reverse	CTT CTT GAT AGG TTT ATG GGT TGG GC
Ckb DAR 1	**chr12:111672017-111672490**
Ckb DAR 1 forward	GAC CTG CAA ACC ACT TGG GAC C
Ckb DAR 1 reverse	GAT GCG CTG GAC TCT GAC GAT G
Ckb DAR 2	**chr12:111670556-111670909**
Ckb DAR 2 forward	CAG ACG CCA AAG AGA CTG GTC A
Ckb DAR 2 reverse	CCT AGT TTG TGC AGT GCC TCC C
Alpl DAR 1	**chr4:111672017-111672490**
Alpl DAR 1 forward	CAC TGG CCC CAT ACT TCA GGG T
Alpl DAR 1 reverse	AGG CTT CTC AGC TCT TGG GGA G
Alpl DAR 2	**chr4:111670556-111670909**
Alpl DAR 2 forward	GGG CAG AAG GCT TGT CCA AGG G
Alpl DAR 2 reverse	GAG CTG ACA TGT CCA GAA AAG AGC
Alpl DAR 3	**chr4:111672017-111672490**
Alpl DAR 3 forward	TTC TTC TCA CAA GCC CCA GGC
Alpl DAR 3 reverse	AGG CTC CCC GGT GAC CTT TTA G
Alpl DAR 4	**chr4:111670556-111670909**
Alpl DAR 4 forward	GGC TGC CAG AGC TGG AAA TCA G
Alpl DAR 4 reverse	AAC CCA TGA GGC TGT CTG GTG T
Alpl DAR 5	**chr4:111672017-111672490**
Alpl DAR 5 forward	GTC ACT GAC CCA GCA ACT ATA GCC
Alpl DAR 5 reverse	TCT AGG GTC AAT ATC CTG CCC A
Alpl DAR 6	**chr4:111670556-111670909**
Alpl DAR 6 forward	TTG CTG TCC CAT CTG TAC CTG A
Alpl DAR 6 reverse	TGG TGA CCT CTT AAC AAC AGA GTG GC
Alpl DAR 7	**chr4:111672017-111672490**
Alpl DAR 7 forward	AGG CCA CTC ACC TCT GCA GTC
Alpl DAR 7 reverse	GGA GGG TGG AGC TAG GGG TGA T
Alpl DAR 8	**chr4:111670556-111670909)**
Alpl DAR 8 forward	TCC CCT CCC TTT TGC TTT GTT TC
Alpl DAR 8 reverse	GCA TTT CAA GGT GGC AGC CAG A
