# Supplementary material for: ADRA1A–Gαq signalling potentiates adipocyte thermogenesis through CKB and TNAP
Source: Nat Metab. 2022 Nov 7;4(11):1459–73. doi: 10.1038/s42255-022-00667-w (PMC9684074; doi:10.1038/s42255-022-00667-w)
Supplement: Supplementary file 2 — Reporting Summary [file 42255_2022_667_MOESM2_ESM.pdf]

nature portfolio

Corresponding author(s): Lawrence Kazak

Last updated by author(s): Sep 14, 2022

Reporting Summary

Nature Portfolio wishes to improve the reproducibility of the work that we publish. This form provides structure for consistency and transparency in reporting. For further information on Nature Portfolio policies, see our Editorial Policies and the Editorial Policy Checklist.

Statistics

For all statistical analyses, confirm that the following items are present in the figure legend, table legend, main text, or Methods section.

n/a	Confirmed	
☐	☒	The exact sample size (n) for each experimental group/condition, given as a discrete number and unit of measurement
☐	☒	A statement on whether measurements were taken from distinct samples or whether the same sample was measured repeatedly
☐	☒	The statistical test(s) used AND whether they are one- or two-sided<br>*Only common tests should be described solely by name; describe more complex techniques in the Methods section.*
☒	☐	A description of all covariates tested
☐	☒	A description of any assumptions or corrections, such as tests of normality and adjustment for multiple comparisons
☐	☒	A full description of the statistical parameters including central tendency (e.g. means) or other basic estimates (e.g. regression coefficient) AND variation (e.g. standard deviation) or associated estimates of uncertainty (e.g. confidence intervals)
☐	☒	For null hypothesis testing, the test statistic (e.g. F, t, r) with confidence intervals, effect sizes, degrees of freedom and P value noted<br>*Give P values as exact values whenever suitable.*
☒	☐	For Bayesian analysis, information on the choice of priors and Markov chain Monte Carlo settings
☒	☐	For hierarchical and complex designs, identification of the appropriate level for tests and full reporting of outcomes
☐	☒	Estimates of effect sizes (e.g. Cohen's d, Pearson's r), indicating how they were calculated

Our web collection on statistics for biologists contains articles on many of the points above.

Software and code

Policy information about availability of computer code

Data collection	RNA-sequencing: Illumina NovaSeq S4 lane using Xp protocol<br>ATAC-sequencing: HiSeq (Michael Smith Genome Sciences Centre, BC Cancer Research Institute)<br>QPCR: CFX Maestro 2017<br>Adipocyte respiration: Rank Brothers Dual Digital model 20: Picolog 6 data logging software<br>Indirect calorimetry: Sable Systems International, Promethion high-definition behavioural phenotyping system data acquisition software (IM-3 v.20.0.3).
Data analysis	RNA sequencing analysis: STAR (v.2.0.2), Trimmomatic (v.0.36), RTA (v3.4.4), bcl2fastq2 (v2.20), HTSeq (v.0.6.0), edgeR's TMM algorithm (v3.26.8)<br>ATAC-sequencing analysis: Trimmomatic (v.0.36), BWA-MEM (v.0.7.12), Picard tools (v.2.0.1), MACS2 (v.2.1.1.20160309), HOMER (v.4.9.1), limma R package (v.3.40.6)<br>Sable Systems International MacroInterpreter software (v.2.41) using One-Click Macro (v.2.37).<br>GraphPad Prism, 9<br>Microsoft office Excel<br>ImageJ v.1.44

For manuscripts utilizing custom algorithms or software that are central to the research but not yet described in published literature, software must be made available to editors and reviewers. We strongly encourage code deposition in a community repository (e.g. GitHub). See the Nature Portfolio guidelines for submitting code & software for further information.

Data

Policy information about availability of data

All manuscripts must include a data availability statement. This statement should provide the following information, where applicable:

- Accession codes, unique identifiers, or web links for publicly available datasets
- A description of any restrictions on data availability
- For clinical datasets or third party data, please ensure that the statement adheres to our policy

The RNA-sequencing and ATAC-sequencing data discussed in this publication have been deposited in NCBI's Gene Expression Omnibus and are accessible through GEO SuperSeries accession number GSE207342 (https://www.ncbi.nlm.nih.gov/geo/query/acc.cgi?acc=GSE207342). Source data are provided with this paper.

Field-specific reporting

Please select the one below that is the best fit for your research. If you are not sure, read the appropriate sections before making your selection.

☒ Life sciences ☐ Behavioural & social sciences ☐ Ecological, evolutionary & environmental sciences

For a reference copy of the document with all sections, see nature.com/documents/nr-reporting-summary-flat.pdf

Life sciences study design

All studies must disclose on these points even when the disclosure is negative.

Sample size	Sample sizes were predetermined based on effect size, standard deviation, and significance level required to attain statistical significance of p<0.05 with a 90% probability on the basis of previous experiments using similar methodologies and were deemed sufficient to account for any biological/technical variability (PMIDs: 31161155, 30078553, 28844881, 24439384). For experiments without predetermination, sample sizes were chosen on the basis of prior experience and published standards in the field (see PMIDs above). Sample sizes are indicated for each experiment in the manuscript.
Data exclusions	No data were excluded
Replication	All attempts of replication were successful. Sample sizes for each experiment are reported in the figure legends. Western blots represent independent biological samples.
Randomization	For in vivo studies, mice in each genotype were randomly assigned to treatment groups. For respirometry experiments, samples were processed in random order across multiple oxygen electrodes. For metabolic cage experiments, mice of different genotypes were housed in alternate cages and equally distributed among thermal cabinets to avoid any systematic bias of external environment.
Blinding	Experimenters were not blinded to experimental conditions, because data collection on indirect calorimetry, respirometry, RNA-sequencing, ATAC-sequencing and RT-qPCR is automated and confers high objectivity.

Reporting for specific materials, systems and methods

We require information from authors about some types of materials, experimental systems and methods used in many studies. Here, indicate whether each material, system or method listed is relevant to your study. If you are not sure if a list item applies to your research, read the appropriate section before selecting a response.

Materials & experimental systems

n/a	Involved in the study	
☐	☒	Antibodies
☐	☒	Eukaryotic cell lines
☒	☐	Palaeontology and archaeology
☐	☒	Animals and other organisms
☐	☒	Human research participants
☒	☐	Clinical data
☒	☐	Dual use research of concern

Methods

n/a	Involved in the study	
☒	☐	ChIP-seq
☒	☐	Flow cytometry
☒	☐	MRI-based neuroimaging

Antibodies

Antibodies used	VCL (Cell Signaling; cat. no. 13901; clone E1E9V): diluted at 1:5,000<br>CKB (Abclonal; cat. no. ab12631): diluted at 1:1,000<br>UCP1 (Abcam; cat. no. ab10983): diluted at 1:2,000<br>TNAP 549 (R&D; cat. no. AF2910): diluted at 1:200

	ERRa (Abcam; cat. no. ab76228): diluted at 1:1,000<br>GFP (Abclonal; cat. no. ab290; 550): diluted at 1:1,000<br>HA-Tag (Cell Signaling; cat. no. C29F4): diluted at 1:1,000<br>Anti-rabbit (Promega; cat. no. W401B): diluted at 1:10,000 (v/v) in TBS-T containing 5% milk<br>Anti-mouse (Promega; cat. no. W402B): diluted at 1:10,000 (v/v) in TBS-T containing 5% milk<br>Anti-goat (Promega; cat. no. V805A): diluted at 1:10,000 (v/v) in TBS-T containing 5% milk
Validation	All antibodies are commercial in origin. Validation statements can be found on the manufacturer's website for the following:<br><br>VCL (Cell Signaling; Cat. No. 13901; clone E1E9V): https://www.cellsignal.com/products/primary-antibodies/vinculin-e1e9v-xp-rabbit-mab/13901<br>CKB (Abclonal; Cat. No. ab1263): https://abclonal.com/catalog-antibodies/CKBPolyclonalAntibody/A12631<br>UCP1 (Abcam; Cat. No. ab10983): https://www.abcam.com/ucp1-antibody-ab10983.html<br>TNAP 549 (R&D; cat. no. AF2910): https://www.rndsystems.com/products/mouse-alkaline-phosphatase-alpl-antibody_af2910<br>ERRa (Abcam; cat. no. ab76228): https://www.abcam.com/estrogen-related-receptor-alpha-antibody-epr46y-ab76228.html<br>GFP (Abclonal; cat. no. ab290): https://www.abcam.com/gfp-antibody-ab290.html<br>HA-Tag (Cell Signaling; cat. no. C29F4): https://www.cellsignal.com/products/primary-antibodies/ha-tag-c29f4-rabbit-mab/3724

Eukaryotic cell lines

Policy information about cell lines

Cell line source(s)	Immortalized brown adipocytes generated in-house
Authentication	Immortalized brown adipocytes were authenticated based on morphology following exposure to an adipogenic cocktail. This indicated that the cells differentiated into lipid-laden fat cells.
Mycoplasma contamination	Cell lines were not tested for mycoplasma.
Commonly misidentified lines<br>(See ICLAC register)	No commonly misidentified cells were used in this study.

Animals and other organisms

Policy information about studies involving animals; ARRIVE guidelines recommended for reporting animal research

Laboratory animals	Mouse experiments were performed according to procedures approved by the Animal Resource Centre at McGill University and complied with guidelines set by the Canadian Council of Animal Care. The photoperiod was fixed at a 12-h light/ 12-h dark schedule (light 0700 to 1900) with lights on at 0700 hours being defined as Zeitbeger time 0 (ZT0). Mice had ad libitum access to drinking water and a low fat diet (3.1 kcal/g energy density) with 24%, 16%, and 60% of Calories from protein, fat, and carbohydrate, respectively (2920X, Envigo, Madison, WI, USA). All mice were born and housed in groups (3-5 mice per cage) at 22ºC ±2ºC at 30-40% humidity with bedding and shredded paper strips in the cage until experimental intervention (6-9 weeks of age). Suitable housing temperature of mice to optimally mimic human physiology is disputed. We followed the suggestion that when provided with bedding and nesting materials standard room temperature (22-24ºC) is appropriate46. For cold exposure experiments, mice were singly housed in cages with bedding and shredded paper strips and with ad libitum access to drinking water and a low fat diet. Mouse experiments used age-matched littermates and were conducted at the temperature indicated in each figure legend. Mice were killed by cervical dislocation and tissues were immediately flash-frozen in liquid nitrogen and store at -80˚C until further analysis. Wild-type C57BL/6N mice were purchased from Charles River (strain code: 027). Ckbfl/fl mice were previously described2. Ucp1CreERT2 mice47 were bred to Ppargc1afl/fl mice to generate experimental groups (Ppargc1afl/fl and Ppargc1aUcp1CreERT2). Inducible BAT-specific overexpression of Gpr3 (I-3BO) and control animals have been previously described12. AdipoqCre mice (B6;FVB-Tg(AdipoQ-Cre)1Evdr/J), stock 028020), maintained on a C57BL/6J background, were bred to (Ckbfl/fl)2, (Ebf1/2fl/fl)16, and (Esrra/gfl/fl)17,48,49 mice to generate experimental groups (Ebf1/2fl/fl and Ebf1/2AdipoqCre)16 (Esrra/gfl/fl and Esrra/gAdipoqCre) (Ckbfl/fl and CkbAdipoqCre). LSL-hM3Dq-DREADD mice (B6N;129-Tg(CAG-CHRM3*,-mCitrine)1Ute/J), stock 026220) were bred to AdipoqCre mice to generate mice that conditionally express a HA-tagged modified muscarinic receptor (HA-hM3Dq) selectively in adipocytes (hM3DqAdipoqCre). hM3DqAdipoqCre mice were crossed with Ckbfl/fl mice to generate hM3DqAdipoqCre:Ckb+/+, hM3DqAdipoqCre:Ckbfl/+ or hM3DqAdipoqCre:Ckbfl/fl mice. Sex of mice used for experiments is noted in the Figure legends an in the source data.
Wild animals	The study did not involve wild animals.
Field-collected samples	The study did not involve samples collected from the field.
Ethics oversight	Animal experiments were performed according to procedures approved by the Animal Resource Centre at McGill University and complied with guidelines set by the Canadian Council of Animal Care.

Note that full information on the approval of the study protocol must also be provided in the manuscript.

Human research participants

Policy information about studies involving human research participants

Population characteristics	First cohort – Joslin Diabetes Center adipose tissue cohort: Details on procedures of human subject adipose tissue biopsy collection have been described previously59,60. Briefly, ten paired human neck fat samples were obtained from superficial

subcutaneous adipose tissue (SAT) depots and deep BAT tissue located proximal to the carotid sheath (n = 10 for each tissue; 5 males and 5 females). Sex was determined based on self-reporting. These patient volunteers (age 49 ± 12.6 years) were typically being treated for cervical spine stenosis, causing radiculopathy or myelopathy. The patients did not undergo metabolic or physiological testing in conjunction with their spine treatment. Tissue processing, RNA isolation, and analysis of gene expression has been previously described59. Briefly, analysis of gene expression using GeneChIP PrimeView (Affymetrix, Santa Clara, CA) was performed on matched biopsies as previously described60. RNA was isolated from clonal cell lines using Direct-zol RNA MiniPrep kit (Zymo Research, Irvine, CA) according to the manufacturer's instructions. The quality of total RNA was evaluated by A260/A280 ratio, which was within the value of 1.9 to 2.0 defined as high quality total RNA. Biotin-labeled cRNA was synthesized, purified and fragmented using GeneChip 3'IVT Express Kit (Affymetrix, Santa Clara, CA). Integrity and fragmented cRNA was assessed by running aliquots on the Agilent 2100 Bioanalyzer prior to proceeding further. The high quality cRNA meets the following criteria: the A260/A280 ratio should fall within the value of 1.9 to 2.0; the 28S/18S RNA bands (from the gel) should be crisp and the intensity of the 28S band should be roughly twice the intensity of the 18S band. Array hybridization and scanning were performed by the Advanced Genomics and Genetics Core of Joslin Diabetes Center according to established methods. Microarray data were normalized using robust multi-array average (RMA), which placed it on a log-2 scale. All subjects gave informed consent prior to taking part in the study. This study followed the institutional guidelines of and was approved by the Human Studies Institutional Review Boards of Beth Israel Deaconess Medical Center and Joslin Diabetes Center

Second cohort – UTMB Washington University adipose tissue cohort: 6 men and 17 women (n=23) with overweight or obesity (age 41 ± 12 years, BMI 31.0 ± 3 kg/m2) were enrolled in two clinical trials (NCT02786251 and NCT01791114) performed to determine the role of BAT in metabolic regulation in people. Self-reported sex was the same with that assigned at birth. None of the participants had past medical history of gender reassignment hormone therapy or surgery. All participants completed a comprehensive screening evaluation that included a medical history and physical examination, standard blood tests, and an oral glucose tolerance test. Potential participants were excluded if they had diabetes or other serious diseases, smoked cigarettes, consumed excessive alcohol, were pregnant or lactating, or had metal implants that interfered with the imaging procedures. The studies were approved by the Institutional Review Board of the University of Texas Medical Branch (UTMB) in Galveston and the Washington University School of Medicine in St. Louis. Written informed consent was obtained from all subjects before their participation. Each participant completed a cold exposure study visit to assess BAT volume and activity and to obtain supraclavicular adipose tissue biopsies. During this visit, a standard cooling protocol was performed to maximize non-shivering thermogenesis61,62. After 6 hours of mild exposure to cold (~20oC), an 18F-fluoro-deoxy-glucose (18F-FDG)-positron emission tomography-computed tomography (PET/CT) scan was performed to determine BAT characteristics (volume and activity)61. Adipose tissue samples from the supraclavicular area - where BAT is primary localized in humans - obtained using a PET/CT-guided percutaneous needle biopsy technique63.
Adipose tissue processing and RNA sequencing analysis: Approximately 100 mg of adipose tissue was used for extraction of RNA using the RNeasy Lipid Tissue Mini Kit (Qiagen Inc., Valencia, CA) including an on-column DNAse digestion step. RNA sequencing libraries were generated using the Illumina TruSeq Stranded Total RNA Library Prep Gold with TruSeq Unique Dual Indexes (Illumina, San Diego, CA). Samples were processed following manufacturer's instructions, except modifying RNA shear time to five minutes. Resulting libraries were multiplexed and sequenced with 75 base pair (bp) single reads (SR75) to a depth of approximately 25 million reads per sample on an Illumina HiSeq 4000. Samples were demultiplexed using bcl2fastq v2.20 Conversion Software (Illumina, San Diego, CA).

Third cohort – Danish adult neck adipose tissue cohort: Adipose tissue biopsies from the superficial (subcutaneous and subplatysmal) neck fat and deep (carotid sheath, longus colli, and prevertebral) neck fat were collected during surgery, where covariate-relevant populations have been described (70). None of the subjects had diabetes nor were they administered β-adrenergic antagonists. All biopsies were collected during winter and early spring and were instantly frozen in liquid nitrogen. Only paired biopsies from SAT and BAT of the same subjects were used for associations (n = 73). All study participants gave informed written consent. The study was approved by the Central Denmark Region ethics committee and was performed in accordance with the Declaration of Helsinki. CKB, ALPL, ADRA1A, and UCP1 mRNA expression was analyzed using RT-qPCR as described above.

Recruitment

First cohort: Healthy volunteers were recruited via electronic advertisements.
Second cohort: Subjects were enrolled in two clinical trials (NCT02786251 and NCT01791114).
Third cohort: Patients admitted for elective neck surgery at Aarhus University Hospital for various malignant and benign conditions such as thyroid cancer, thyroid adenomas, hyperthyroidism and primary hyperparathyroidism were recruited.

Ethics oversight

First cohort: This study followed the institutional guidelines and was approve
d by the Human Studies Institutional Review Boards of Beth Israel Deaconess Medical Center and Joslin Diabetes Center
Second cohort: The studies were approved by the Institutional Review Board of the University of Texas Medical Branch (UTMB) in Galveston and the Washington University School of Medicine in St. Louis. Written informed consent was obtained from all subjects before their participation.
Third cohort: The study was approved by the Central Denmark Region ethics committee and was performed in accordance with the Declaration of Helsinki. All study participants gave informed written consent.

Note that full information on the approval of the study protocol must also be provided in the manuscript.
